# EXAFS and XANES analysis of oxides at the nanoscale

**DOI:** 10.1107/S2052252514021101

**Published:** 2014-10-31

**Authors:** Alexei Kuzmin, Jesús Chaboy

**Affiliations:** a Institute of Solid State Physics, University of Latvia, LV-1063 Riga, Latvia; b Instituto de Ciencia de Materiales de Aragón, Consejo Superior de Investigaciones Científicas and Departamento de Física de la Materia Condensada, Universidad de Zaragoza, 50009 Zaragoza, Spain

**Keywords:** EXAFS, XANES, oxide nanomaterials, nanocrystalline materials

## Abstract

Worldwide research activity at the nanoscale is triggering the appearance of new, and frequently surprising, materials properties in which the increasing importance of surface and interface effects plays a fundamental role. This opens further possibilities in the development of new multifunctional materials with tuned physical properties that do not arise together at the bulk scale. Unfortunately, the standard methods currently available for solving the atomic structure of bulk crystals fail for nanomaterials due to nanoscale effects (very small crystallite sizes, large surface-to-volume ratio, near-surface relaxation, local lattice distortions *etc*.). As a consequence, a critical reexamination of the available local-structure characterization methods is needed. This work discusses the real possibilities and limits of X-ray absorption spectroscopy (XAS) analysis at the nanoscale. To this end, the present state of the art for the interpretation of extended X-ray absorption fine structure (EXAFS) is described, including an advanced approach based on the use of classical molecular dynamics and its application to nickel oxide nanoparticles. The limits and possibilities of X-ray absorption near-edge spectroscopy (XANES) to determine several effects associated with the nanocrystalline nature of materials are discussed in connection with the development of ZnO-based dilute magnetic semiconductors (DMSs) and iron oxide nanoparticles.

## Abstract 2

This work presents a discussion of the possibilities offered by X-ray absorption spectroscopy (XAS) to study the local structure of nanomaterials. The current state of the art for the interpretation of extended X-ray absorption fine structure (EXAFS), including an advanced approach based on the use of classical molecular dynamics, is described and exemplified in the case of NiO nanoparticles. In addition, the limits and possibilities of X-ray absorption near-edge spectroscopy (XANES) in determining several effects associated with the nanocrystalline nature of materials are also discussed in connection with the development of ZnO-based dilute magnetic semiconductors and iron oxide nanoparticles.

## 1. Introduction   

Nanomaterials are of fundamental and technological importance as they have fascinating physical and chemical properties which can be exploited for numerous applications (Gleiter, 1989 ▶, 1995 ▶; Fernández-García *et al.*, 2004 ▶; Goesmann & Feldmann, 2010 ▶). Since nanomaterial properties depend strongly on size and shape, a key requirement for understanding and controlling them is knowledge of the atomic structure. This is a challenging task, the solution of which requires a complex approach based on the use of different complementary experimental techniques (Rao & Biswas, 2009 ▶) and advanced computational methods (Billinge & Levin, 2005 ▶).

X-ray absorption spectroscopy (XAS) is one of the direct structural probes providing information on the local environment around a photoabsorber (Lee *et al.*, 1981 ▶; Rehr & Albers, 2000 ▶; Aksenov *et al.*, 2006 ▶; Yano & Yachandra, 2009 ▶; Li *et al.*, 2010 ▶; Boscherini, 2013 ▶). XAS is an excellent tool for this purpose, because it can be applied equally successfully to both ordered and disordered materials. XAS is also element-selective, and is sensitive to high dilutions, and to length scales down to nanoparticles (NPs) and even molecules. XAS has gained in popularity during the past decade with progress in synchrotron radiation sources, which ensure a high quality of experimental data (Pettifer *et al.*, 2007 ▶; Purans *et al.*, 2008 ▶) and open new horizons for studies that are time-dependent (Oguz Er *et al.*, 2012 ▶) or under extreme conditions (Di Cicco *et al.*, 2011 ▶). The spatial resolution of XAS experiments has been pushed down to the nanoscale using nanofocused X-ray beams (Martínez-Criado *et al.*, 2012 ▶, 2013 ▶), or by combining XAS with scanning probe microscopy (Larcheri *et al.*, 2008 ▶) and scanning transmission X-ray microscopy (Guttmann *et al.*, 2012 ▶).

The application of XAS to nanomaterials represents a rapidly growing field of research, and several review papers have been published on this topic in the last decade (Modrow, 2004 ▶; Frenkel *et al.*, 2011 ▶; Mino *et al.*, 2013 ▶). Despite the great deal of research performed to date, the problem of determining coordination numbers in nanocrystalline metals and oxides, an early problem in catalysis (Díaz-Moreno *et al.*, 1997 ▶), still persists today, with papers both for and against the presence of highly disordered interfaces and grain boundary regions (Chadwick *et al.*, 2003 ▶; Stern *et al.*, 1995 ▶; Boscherini *et al.*, 1998 ▶; Baker *et al.*, 2009 ▶; Dubiel *et al.*, 2000 ▶). Most research activities are concentrated on the analysis of a few nearest coordination shells around a photoabsorber and many of them are dedicated to metallic NPs, because the influence of under-coordinated atoms at the surface and the limited size of the NPs modify significantly the atomic structure and lattice dynamics of NPs, thus making reliable analysis of the XAS spectra a complicated task. Nevertheless, methods to characterize the structure of metallic NPs from an analysis of the nearest coordination shells and to determine the mean crystallite size have been proposed (Calvin *et al.*, 2005 ▶; Frenkel, 2007 ▶). At the same time, in a recent detailed study of metal NPs (Agostini *et al.*, 2014 ▶), the authors emphasized the need to use complementary techniques to extract reliable structural information. In particular, they showed that a combination of transmission electron microscopy (TEM), chemisorption measurements and extended X-ray absorption fine structure (EXAFS) analysis up to the fourth coordination shell, with Monte Carlo simulations, is required to account properly for the size distribution of metal NPs, which influences significantly the average values of the structural parameters (Agostini *et al.*, 2014 ▶).

To date, the EXAFS region of the X-ray absorption spectrum has attracted most attention in the field. However, recent works have proposed that several effects associated with the change in size scale, such as the occurrence of charge-transfer effects under the influence of ligands or the existence and type of vacancies, can be probed from the study of the X-ray absorption near-edge spectroscopy (XANES) part of the spectrum (Ma *et al.*, 2012 ▶; Ciatto *et al.*, 2011 ▶).

In this paper, we will describe the present state of the art for the interpretation of EXAFS, including an advanced approach based on the use of classical molecular dynamics and its application to nickel oxide NPs. The limits and possibilities of XANES for determining several effects associated with the nanocrystalline nature of the materials will be discussed in connection with the development of ZnO-based dilute magnetic semiconductors (DMSs) and iron oxide NPs.

## 2. EXAFS spectroscopy of nanomaterials   

### 2.1. EXAFS within the multiple-scattering approximation   

**Figure 1:**
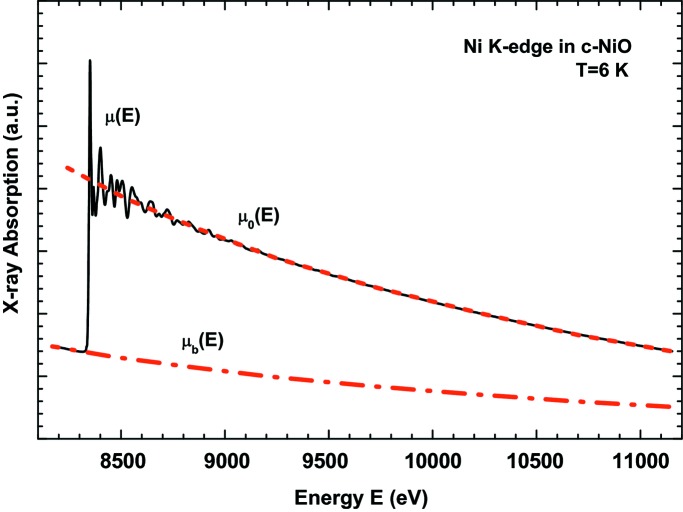
Low-temperature (6 K) X-ray absorption spectrum of bulk NiO at the Ni *K*-edge (solid line). The background μ_b_(*E*) and atomic-like μ_0_(E) absorption contributions are shown by dash-dotted and dashed lines, respectively.

The EXAFS χ^*l*^(*k*) past the absorption edge of orbital type *l* is defined as (Fig. 1 ▶) (Lee *et al.*, 1981 ▶)
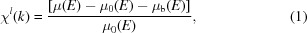
where μ(*E*) is the experimentally measured X-ray absorption coefficient, μ_b_(*E*) is the background absorption and μ_0_(*E*) is the atomic-like absorption due to an isolated absorbing atom. The excited photoelectron wavenumber *k* is related to its energy *E* by 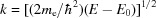, where *m*_e_ is the electron mass, ℏ is the Planck constant and *E*_0_ is the threshold energy, *i.e.* the energy of a free electron with zero momentum.

Within the multiple-scattering (MS) theory, the EXAFS χ^*l*^(*k*) can be decomposed into a series (Ruiz-Lopez *et al.*, 1988 ▶; Brouder *et al.*, 1989 ▶; Rehr & Albers, 2000 ▶)
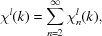


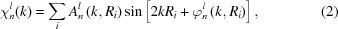
where 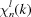 includes contributions from the (*n*−1)-order scattering processes of the excited photoelectron by the neighbouring atoms, before it returns to the photoabsorber. While the series is infinite, in many practical cases only the first few terms produce a significant contribution to the total EXAFS, due to the finite lifetime of the excitation, the scattering path lengths and cancellation effects.

**Figure 2:**
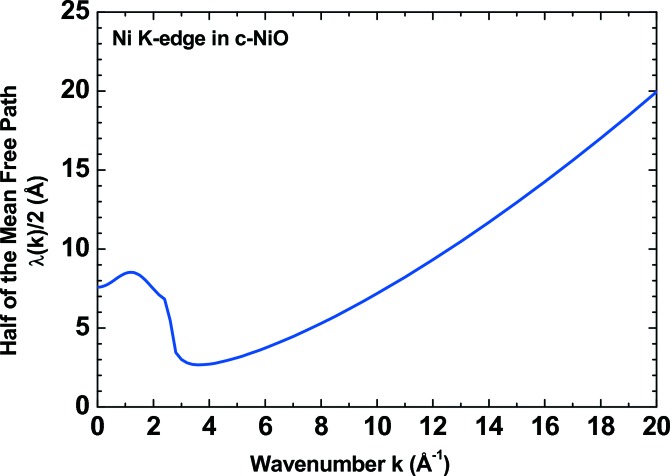
The mean free path λ(*k*) of a photoelectron plotted *versus* wavenumber *k* for the Ni *K*-edge, including the core–hole effect.

Equation (2) can be rewritten in a more conventional way (Zabinsky *et al.*, 1995 ▶; Rehr & Albers, 2000 ▶; Rehr *et al.*, 2009 ▶) as
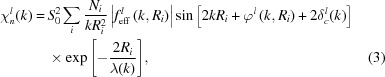
where 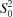 is a many-body reduction factor accounting for amplitude damping due to multi-electron effects (intrinsic losses), *N*_*i*_ is the degeneracy of the photoelectron scattering path, *R*_*i*_ is the effective path half-length, 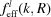 is the photoelectron effective scattering amplitude for path *i*, ϕ^*l*^(*k*, *R*) is the phase shift function for path *i*, 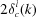 is the final-state phase shift at the central (absorbing) atom and λ(*k*) is the energy-dependent mean free path of the photoelectron, which ranges from a few ångströms to ∼10–20 Å (Fig. 2 ▶). Note that, in the case of single scattering processes, *N*_*i*_ has the meaning of the coordination number and *R*_*i*_ equals the radius of the *i*-th coordination shell.

The time-scale of the photoabsorption process is about 10^−15^–10^−16^ s, which is significantly shorter than the characteristic time (∼10^−13^ s) of thermal vibrations. Therefore, the atoms may be considered as frozen at their instantaneous positions during a single excitation process, and the total EXAFS corresponds to the configurational average of all atomic positions. The effect of thermal disorder is conventionally introduced (Beni & Platzman, 1976 ▶) within the harmonic approximation, which adds an exponential term exp(−2σ^2^ *k* ^2^) into equation (3),[Disp-formula fd3]

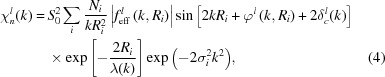
where σ^2^ is the Debye–Waller factor or mean-square relative displacement (MSRD). This factor is responsible for the exponential damping of the EXAFS amplitude with increasing energy (wavenumber) and for its temperature dependence. It can be left as a free parameter during the EXAFS simulation or evaluated using semi-empirical correlated Einstein or Debye models (Sevillano *et al.*, 1979 ▶; Beni & Platzman, 1976 ▶; Vaccari & Fornasini, 2006 ▶; Bunker, 2010 ▶), the equation-of-motion method (Poiarkova & Rehr, 1999 ▶) or the more sophisticated first-principles lattice dynamics theory (Dimakis & Bunker, 1998 ▶; Vila *et al.*, 2007 ▶). Finally, the anharmonic correction, required, for example, to describe the effect of thermal expansion on EXAFS (Eisenberger & Brown, 1979 ▶), can be treated within the cumulant expansion technique (Bunker, 1983 ▶; Dalba *et al.*, 1993 ▶; Fujikawa & Miyanaga, 1993 ▶; Fornasini, 2001 ▶).

**Figure 3:**
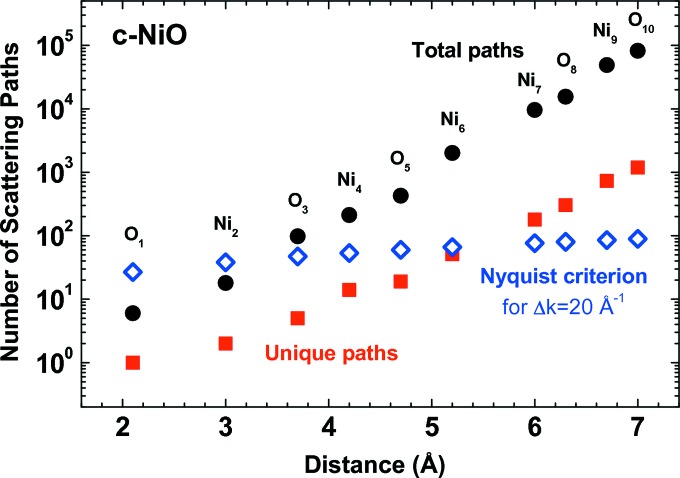
The dependence of the number of scattering paths on cluster size for NiO. Note the logarithmic scale on the vertical axis.

The situation becomes non-trivial in the case of nano­materials, since the relaxation of the atomic structure upon reduction of the NP size can significantly affect their phonon properties (Gouadec & Colomban, 2007 ▶). At the same time, relaxation of the structure and the presence of a surface lead to the appearance of many non-equivalent atomic sites, the number of which will increase even more in the presence of defects (vacancies, impurity atoms). For example, a decrease in the average coordination number and the average nearest-neighbour distance, accompanied by an increase in static disorder, has been observed in rhodium NPs upon reducing their size (Agostini *et al.*, 2013 ▶). This fact makes it impractical to use equation (4) to analyse the full EXAFS signal from a nanomaterial, due to the very large number of fitting parameters required. To illustrate this problem, let us consider the case of bulk nickel oxide with a rock-salt cubic structure (Fig. 3 ▶). It is possible to calculate the total number of scattering paths, the number of unique paths due to the cubic symmetry and the maximum number of fitting parameters *N*_par_ which can be used in the EXAFS model according to the Nyquist theorem [*N*_par_ = 2Δ*k*Δ*R*/π (Bordiga *et al.*, 2013 ▶)], for a relatively long EXAFS signal with Δ*k* = 20 Å^−1^, as a function of radial distance *R* (cluster radius around the photoabsorber). As one can see, the Nyquist criterion is already not satisfied at *R* ≃ 5.5 Å in bulk nickel oxide, and this distance will be significantly reduced in a nanomaterial.

Also, one should point out that equation (4) accounts only for radial disorder, whereas the photoelectron effective scattering amplitude and phase shift functions show a nonlinear angular dependence and are sensitive even to small variations in angle along the scattering path, especially in the case of linear atomic chains (Teo, 1986 ▶; Kuzmin & Purans, 1993 ▶). This problem has been addressed in the past for small disorder using the low-order Taylor expansion for amplitude and phase of the EXAFS signal (Filipponi *et al.*, 1995 ▶; Filipponi & Di Cicco, 1995 ▶).

To overcome the above-mentioned problems, several advanced approaches have been developed in the last 15 years. They are based on calculation of the configuration-averaged EXAFS signal using a set of atomic configurations, which can be obtained from *ab initio* or classical molecular dynamics (MD) (D’Angelo *et al.*, 1994 ▶, 2002 ▶; Merkling *et al.*, 2001 ▶; Cabaret *et al.*, 2001 ▶; Okamoto, 2004 ▶; Farges *et al.*, 2004 ▶; Ferlat *et al.*, 2005 ▶; Kuzmin & Evarestov, 2009 ▶; Price *et al.*, 2012 ▶; Yancey *et al.*, 2013 ▶), Monte Carlo (MC) simulations (Metropolis *et al.*, 1953 ▶) or reverse Monte Carlo (RMC) simulations (Winterer, 2000 ▶; McGreevy, 2001 ▶; Di Cicco & Trapananti, 2005 ▶; Gereben *et al.*, 2007 ▶; Krayzman *et al.*, 2009 ▶; Krayzman & Levin, 2010 ▶; Levin *et al.*, 2014 ▶; Timoshenko, Anspoks *et al.*, 2014*a* ▶,*b* ▶; Timoshenko, Kuzmin & Purans, 2014 ▶). Note that, in the first two methods, MD and MC, a force-field model should be provided to describe the interactions between atoms (Abraham, 1986 ▶), whereas it is not required in the RMC technique (McGreevy & Pusztai, 1988 ▶; Gereben *et al.*, 2007 ▶).

### 2.2. EXAFS from the nearest shells   

The use of classical MD is limited by the simplicity of existing force-field models and is not applicable in situations where the electronic structure of a material leads to a strong local structure distortion such as, for example, in the case of the Jahn–Teller effect (Kunz & Brown, 1995 ▶). In this case, reliable EXAFS analysis is limited to the nearest environment but can still provide useful information on the relaxation of the local atomic structure in NPs.

Since high-order (*n* > 2) scatterings have long path lengths, they can be isolated in real space using the Fourier filtering procedure, and thus the contribution from the nearest atoms containing only single-scattering (*n* = 2) processes can be singled out and analysed separately. This is the most widely used approach to EXAFS analysis, for which the exact analytical expression has been obtained in the past within the curved-wave formalism (Ashley & Doniach, 1975 ▶; Lee & Pendry, 1975 ▶).

In the harmonic approximation for thermal motion, single-scattering EXAFS is given by equation (4), whereas in the case of weak disorder it can be parameterized using the cumulant decomposition (Bunker, 1983 ▶; Dalba *et al.*, 1993 ▶; Fornasini, 2001 ▶) as
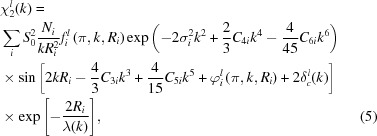
where *C*_3*i*_, *C*_4*i*_, *C*_5*i*_ and *C*_6*i*_ are cumulants of a distribution, taking into account anharmonic effects and/or non-Gaussian disorder, and *f* ^*l*^(π, *k*, *R*_*i*_) and ϕ^*l*^(π, *k*, *R*_*i*_) are the backscattering amplitude of the photoelectron due to the atoms of *i*-th coordination shell.

When the first coordination shell around the photoabsorber is strongly distorted, the cumulant series will not converge. In this case, one should use the EXAFS formula as expressed in terms of the radial distribution function (RDF) 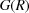 (Lee *et al.*, 1981 ▶),
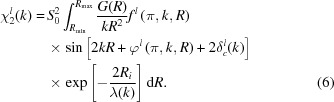
Here, *G*(*R*) corresponds to the number of atoms located in the spherical shell around the photoabsorber between *R* and *R* + d*R*, so that the number *N* of atoms located in the region between *R*_min_ and *R*_max_ is given by the integral 
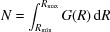
. To determine the RDF *G*(*R*), the regularization technique (Babanov *et al.*, 1981 ▶; Ershov *et al.*, 1981 ▶; Kuzmin & Purans, 2000 ▶) can be used to solve the integral of equation (6) as an ill-posed problem (Bakushinsky & Goncharsky, 1994 ▶). Recent applications of this method to study the local structure in nanosized tungstates (NiWO_4_, CoWO_4_, CuWO_4_, ZnWO_4_) can be found in Kalinko & Kuzmin (2011 ▶) and Anspoks, Kalinko, Timoshenko & Kuzmin (2014 ▶).

### 2.3. Structure relaxation and disorder in nanosized NiO   

The use of classical MD simulations for the interpretation of EXAFS (the MD-EXAFS method) in nanomaterials will be discussed below for the example of Ni *K*-edge EXAFS in nanosized nickel oxide (nano-NiO) (Anspoks *et al.*, 2010 ▶, 2012 ▶; Anspoks & Kuzmin, 2011 ▶; Anspoks, Kalinko, Kalendarev & Kuzmin, 2014 ▶).

**Figure 4:**
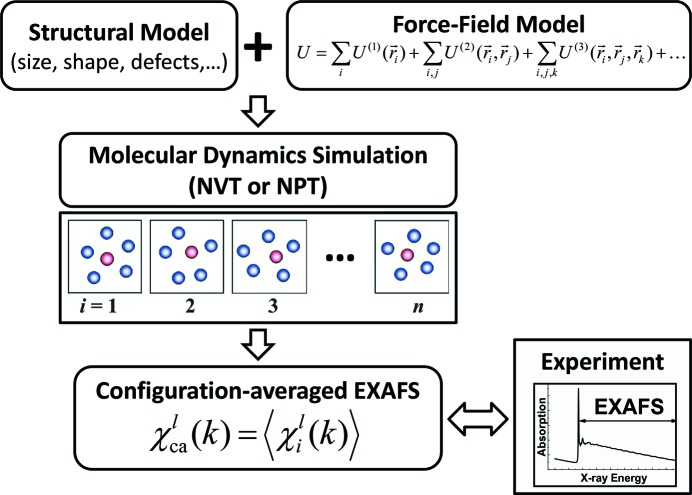
Scheme of the MD-EXAFS calculations.

The general scheme of the MD-EXAFS method is shown in Fig. 4 ▶. First, one needs to define the structural model of the material, *i.e.* the NP chemical composition, shape and size, and to specify the force-field model describing all interactions between atoms. The starting set of force-field parameters can be obtained from their fit to the properties (*e.g.* structure, elastic constants, bulk modulus, phonons) of the nanomaterial bulk counterpart or to a potential energy surface obtained from first-principles calculations (Gale, 1996 ▶). For the next step, the MD simulation is performed using the NVT or NPT ensemble (Abraham, 1986 ▶) for the required temperature and pressure conditions of the EXAFS experiment. At this point, one should take care that the simulation temperature is high enough (near and above the material’s Debye temperature) (Turney *et al.*, 2009 ▶) so that quantum effects can be neglected and classical dynamics is expected to be highly valid. As a result of the MD simulation, a set of instantaneous atomic configurations (‘snapshots’ of the structure) is obtained. These are used to calculate the partial RDFs, with the aim of determining structural parameters such as coordination numbers, interatomic distances and MSRDs for the nearest coordination shells. The values of the structural parameters thus obtained are compared with those determined from conventional analysis of EXAFS, and the agreement between the two sets of data is used as a criterion for adjustment of the force-field model. Such an approach allows one to exclude the unreliable evolution of the nanomaterial structure during the MD run and to reduce the number of time-consuming EXAFS calculations to be performed in the next step. Finally, the configuration-averaged EXAFS 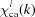 = 〈χ^*l*^(*k*)〉 is calculated for a set of snapshots of instant atomic positions generated by MD simulation. This is done by averaging the EXAFS spectra, evaluated by equation (2) for each snapshot independently, over all photoabsorbers in the NP and over the MD simulation time. The calculation of each EXAFS signal can be performed using an *ab initio* multiple-scattering program [*e.g.* *FEFF* (Ankudinov *et al.*, 1998 ▶; Rehr *et al.*, 2010 ▶), *GNXAS* (Filipponi & Di Cicco, 2000 ▶) or *DL EXCURV* (Tomić *et al.*, 2005 ▶; Gurman *et al.*, 1984 ▶, 1986 ▶)], and the convergence of the configuration-averaged EXAFS should be controlled in each case. The agreement between the calculated and experimental EXAFS is used as a criterion to select the best structural model of the NP. The advantage of this method­ology is that a small number of free parameters are used in the NP model, which are optimized to reproduce the experimental EXAFS, taking into account most or even all multiple-scattering contributions and disorder (thermal and static) effects within the large real-space range.

In the MD-EXAFS method (Anspoks *et al.*, 2010 ▶, 2012 ▶; Anspoks & Kuzmin, 2011 ▶; Anspoks, Kalinko, Kalendarev & Kuzmin, 2014 ▶), the force-field model, which is required to describe the interatomic interactions in nano-NiO, is relatively simple and includes only three free parameters (nanoparticle size *L*, concentration of nickel vacancies *C*_vac_ and effective charge of nickel *Z*_Ni_ ions), other parameters being optimized and fixed at the values for bulk nickel oxide.

Nanocrystalline NiO has the rock-salt structure and is an important technological material used in catalysis, electrochromic devices, gas sensors, resistive and magnetoresistive memory, giant magnetoresistive (GMR) spin valve structures, rechargeable batteries and fuel cells. The performance of all these devices relies largely on the oxide stoichiometry. It is known that NiO is a *p*-type semiconductor, usually having an oxygen excess due to the presence of nickel vacancies (*V*_Ni_) (Sato *et al.*, 1993 ▶; Kohmoto *et al.*, 2001 ▶; Yu *et al.*, 2012 ▶), which strongly influences its electrical (Jang *et al.*, 2009 ▶) and magnetic (Mandal *et al.*, 2009 ▶) properties and structural stability (Jang *et al.*, 2011 ▶).

**Figure 5:**
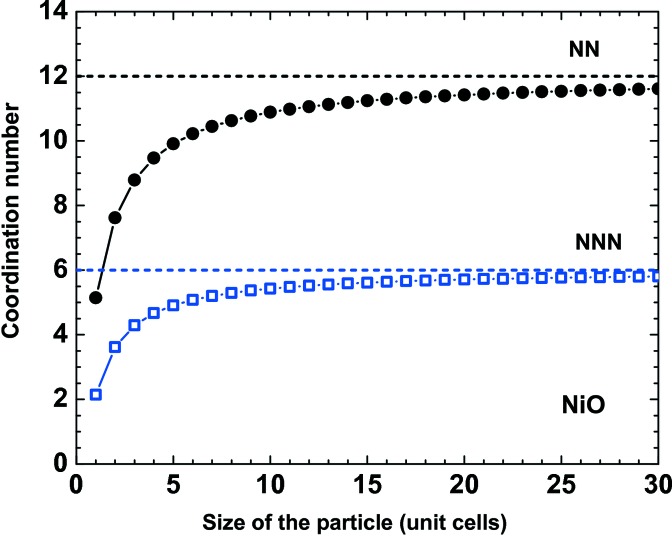
Dependence of the average coordination number for the nearest-neighbour (NN) Ni—Ni_2_ (second shell) and next-nearest-neighbour (NNN) Ni—Ni_4_ (fourth shell) atom pairs on the cubic NiO particle size.

**Figure 6:**
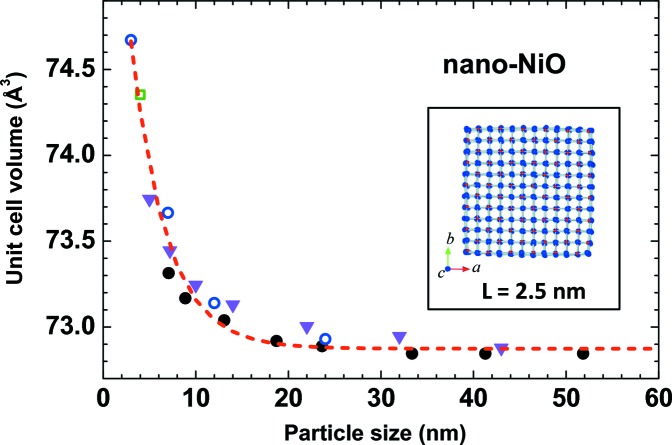
Dependence of the unit-cell volume on NP size. Data are taken from XRD studies, solid circles from Li *et al.* (2006 ▶), empty circles from Ghosh *et al.* (2006 ▶), the empty square from Zheng *et al.* (2008 ▶) and solid triangles from Makhlouf *et al.* (2009 ▶)). The inset shows the NP model with a size *L* = 2.5 nm.

Upon reduction of the crystallite size, the number of under-coordinated atoms located at the surface of the crystallites increases relative to those in the bulk, thus leading to a decrease in the average coordination number (Fig. 5 ▶). Moreover, a bond contraction for under-coordinated atoms at sites surrounding a defect or at the surface of the NP is expected within the bond-order–length–strength (BOLS) correlation mechanism (Sun, 2007 ▶), and should result in an average unit-cell volume compression, commonly observed in metallic NPs (Sun, 2007 ▶). However, a volume expansion has been found in many nanocrystalline metal oxides, for example CeO_2−*x*_ (Tsunekawa *et al.*, 1999 ▶), MgO and α-Fe_2_O_3_ (Fukuhara, 2003 ▶), CaWO_4_ (Li *et al.*, 2007 ▶), BaTiO_3_ (Huang *et al.*, 2007 ▶), CuO (Bianchi *et al.*, 2008 ▶) and rutile TiO_2_ (Kuznetsov *et al.*, 2009 ▶). Such behaviour is well documented in nano-NiO by X-ray diffraction experiments (Li *et al.*, 2006 ▶; Ghosh *et al.*, 2006 ▶; Zheng *et al.*, 2008 ▶; Makhlouf *et al.*, 2009 ▶), which show an increase in unit-cell volume upon crystallite size reduction below ∼20 nm (Fig. 6 ▶).

**Figure 7:**
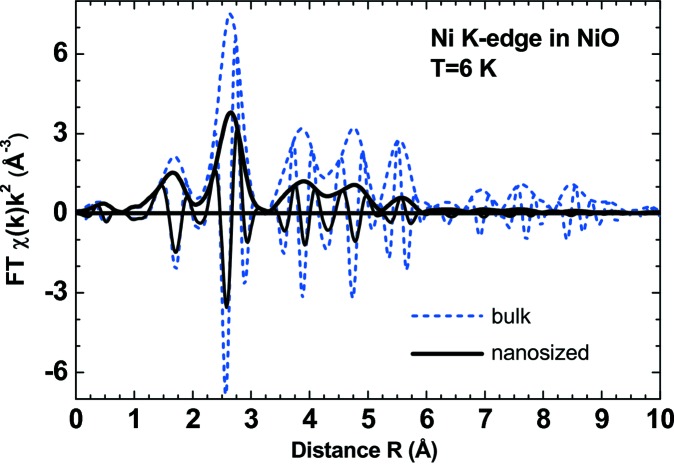
*k* ^2^-weighted phase-uncorrected Fourier transforms (FTs) of the low-temperature (*T* = 6 K) Ni *K*-edge EXAFS spectra in bulk and nanosized NiO (Anspoks *et al.*, 2012 ▶). Both the magnitude and the imaginary parts of the FTs are shown.

**Figure 8:**
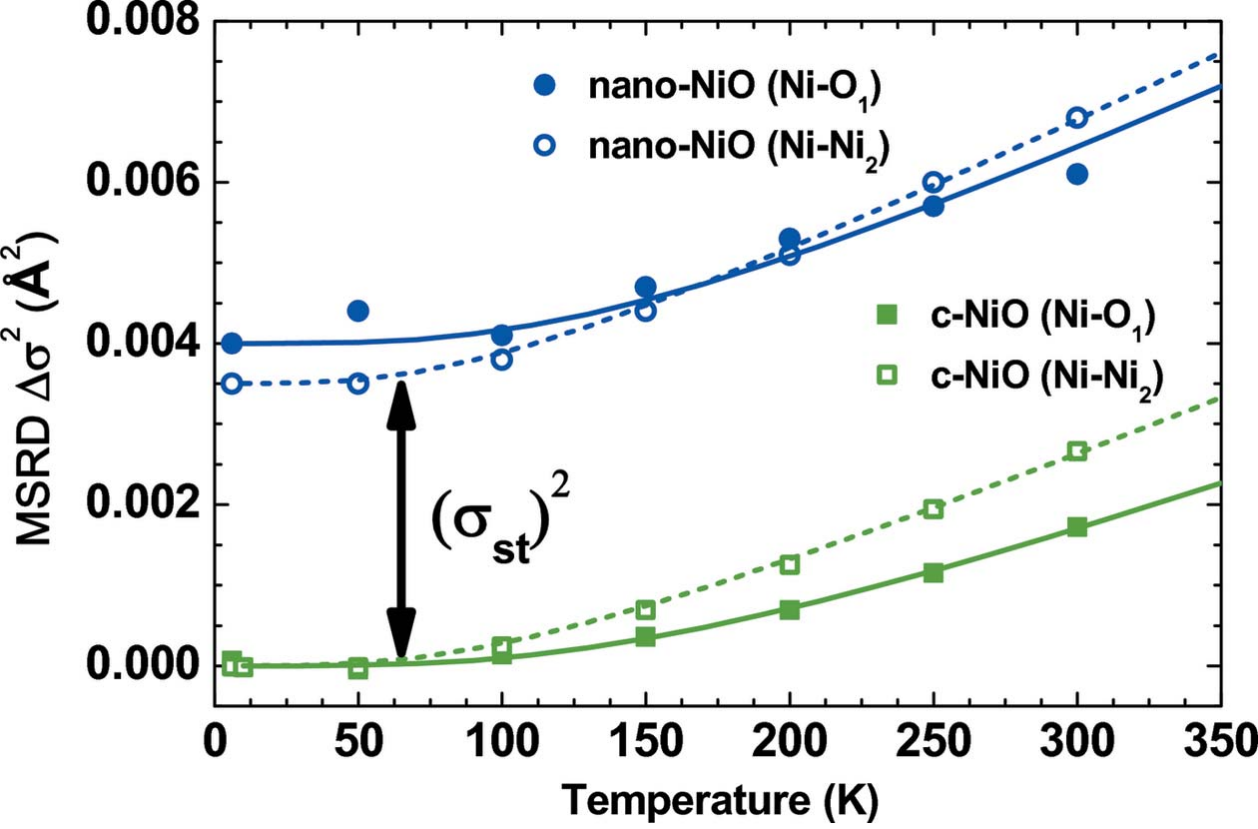
Temperature dependence of the mean-square relative displacements (MSRD) σ^2^ for the first (Ni—O_1_) and second (Ni—Ni_2_) coordination shells in bulk and nanosized NiO, relative to the MSRD value in bulk NiO at *T* = 6 K (Anspoks *et al.*, 2012 ▶). The solid and dashed lines show the correlated Debye models.

EXAFS spectroscopy has been used to study local structure in nickel oxide for a long time. Most works have been dedicated to an investigation of NiO thin films (Kuzmin *et al.*, 1997 ▶; Avendaño *et al.*, 2005 ▶; Jang *et al.*, 2009 ▶, 2011 ▶; Gutiérrez *et al.*, 2013 ▶; Anspoks, Kalinko, Kalendarev & Kuzmin, 2014 ▶; Domínguez-Cañizares *et al.*, 2014 ▶), NiO monolayers on Ag(001) (Luches *et al.*, 2003 ▶; Groppo *et al.*, 2003 ▶, 2004 ▶; Lamberti *et al.*, 2003 ▶; Luches, Groppo *et al.*, 2004 ▶; Lamberti, 2004 ▶) and NPs (Hattori *et al.*, 2002 ▶; Meneses *et al.*, 2007 ▶; Mandal *et al.*, 2009 ▶; Anspoks *et al.*, 2010 ▶, 2012 ▶; Anspoks & Kuzmin, 2011 ▶). One can also mention the work that has been done on ultrathin MgO epilayers on Ag(001) (Luches, D’Addato *et al.*, 2004 ▶), which have a rock-salt crystal structure similar to NiO and thus a closely related behaviour of the EXAFS signal. A comparison of the Fourier transforms of Ni *K*-edge EXAFS for bulk and nanosized NiO is shown in Fig. 7 ▶. The effect of size reduction is clearly observed as a progressive decrease in peak amplitude upon increasing distance. However, even in the nanosized oxide, the structural peaks are well visible up to ∼6 Å, thus making the use of the MD-EXAFS approach feasible. A more detailed analysis of the first two peaks due to the first two coordination shells (Ni—O_1_ and Ni—Ni_2_) indicates that, in the nanosized oxide, the *R*(Ni—Ni_2_) distance increases slightly, whereas the *R*(Ni—O_1_) distance contracts (Anspoks *et al.*, 2012 ▶; Anspoks, Kalinko, Kalendarev & Kuzmin, 2014 ▶). Such a result is not trivial, since in bulk NiO the two distances are proportional: *R*(Ni—Ni_2_) = *R*(Ni—O_1_)(2^1/2^). In addition, it was found that, while the temperature dependence of the MSRD for the Ni—O_1_ and Ni—Ni_2_ atom pairs is close and can be described by the correlated Debye model, there is some additional static disorder (σ_st_)^2^ in nanosized NiO (Anspoks *et al.*, 2012 ▶; Anspoks, Kalinko, Kalendarev & Kuzmin, 2014 ▶) (Fig. 8 ▶). These findings favour the presence of nickel vacancies within the volume of NPs.

The full EXAFS spectrum for NiO NPs has been simulated by Anspoks *et al.* (2012 ▶) using the MD-EXAFS method, starting from the force-field model optimized for bulk NiO by Fisher (2004 ▶). The model includes two-body central force interactions between atoms *i* and *j*, described by the sum of the Buckingham and Coulomb potentials
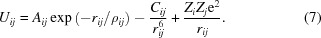
The Buckingham potential parameters *A*, ρ and *C* were fixed to the values found for bulk NiO (Fisher, 2004 ▶; Anspoks *et al.*, 2010 ▶), whereas the charge on the nickel atoms *Z*_Ni_ was used as a free parameter to minimize the residual between the experimental and configuration-averaged EXAFS spectra. The simulations were performed at *T* = 300 K in the canonical ensemble (NVT) using cubic-shaped particles of size *L* × *L* × *L* placed in a large empty box. Nickel vacancies were generated by randomly removing Ni atoms from the model NP, ensuring their homogeneous distribution. Each model NP was characterized by its size *L* and the number of nickel vacancies *N*_vac_. Thus, the vacancy concentration is *C*_vac_ = *N*_vac_/*N*_O_, where *N*_Ni_ and *N*_O_ are the number of nickel and oxygen atoms in the NP, respectively. The charge on the oxygen atoms *Z*_O_ was calculated to maintain electroneutrality of the system, taking into account the number of Ni vacancies as *Z*_O_ = −*Z*_Ni_ *N*_Ni_/*N*_O_. Note that, in this simple model, all Ni ions have the same charge (*Z*_Ni_) and so do all oxygen ions (*Z*_O_).

**Figure 9:**
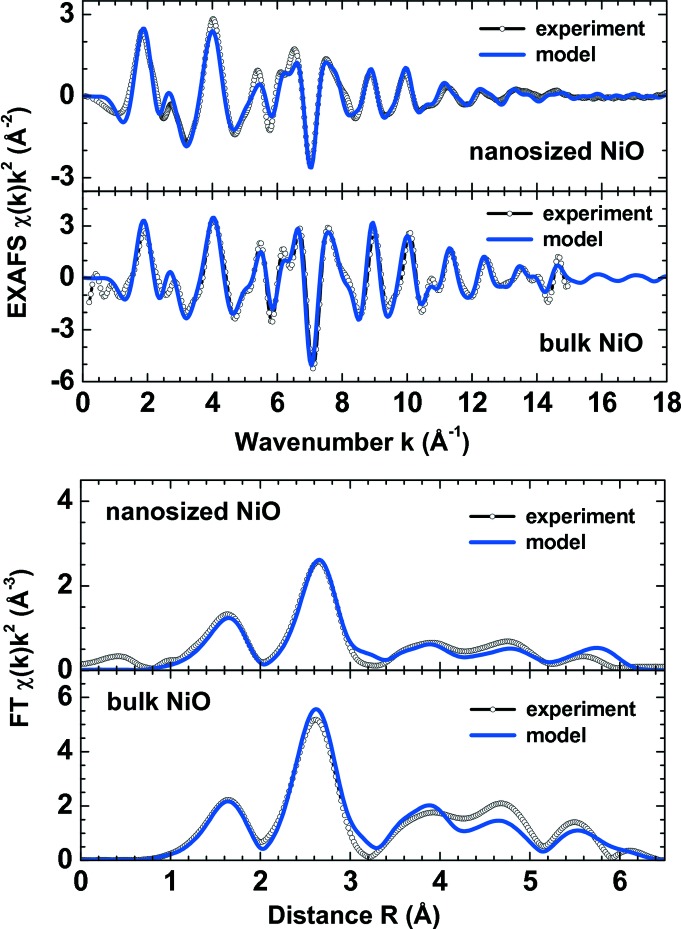
Comparison of experimental (open circles) and configuration-averaged (solid lines) Ni *K-*edge EXAFS χ(*k*)*k* ^2^ and their Fourier transforms for bulk (Anspoks *et al.*, 2010 ▶) and nanosized (Anspoks *et al.*, 2012 ▶) NiO at *T* = 300 K.

An example of the MD-EXAFS simulations for bulk NiO and nanosized NiO (*L* = 1.3–2.1 nm, *C*_vac_ = 1.6–2.0%, *Z*_Ni_ = 1.925) is shown in Fig. 9 ▶. Here, the configuration-averaged Ni *K*-edge EXAFS spectra were calculated using the program *FEFF8* (Ankudinov *et al.*, 1998 ▶), employing a complex exchange and correlation Hedin–Lundqvist potential to account for inelastic effects. As one can see, the MD-EXAFS approach allows one to obtain a good agreement between theory and experiment in both *k*- and *R*-space, taking into account a reasonable NP model, multiple-scattering contributions and thermal and static disorder within a rather simple force-field model. Thus, the method provides a reliable analysis of the nearest and outer coordination shells in a nanomaterial. It can be also used for the development and validation of theoretical models based on experimental EXAFS data.

The weak point of the MD-EXAFS method based on classical MD is the need to have a reliable force-field model and the neglect of quantum effects. As a result, the method cannot be used at low temperatures and will fail to describe the local structure distortion due to electronic effects such as, for instance, in noncentrosymmetric oxides (Halasyamani & Poeppelmeier, 1998 ▶). One can improve the reliability of the method by employing potentially more accurate but computationally much more heavy *ab initio* MD simulations (Spezia *et al.*, 2006 ▶; Pham *et al.*, 2010 ▶; Yancey *et al.*, 2013 ▶). While such an approach allows parameter-free analysis of experimental EXAFS data, also in this case the ability of the method to provide accurate values of the structural parameters will depend on the approximations used, in particular on the type of density functional theory functional (Kalinko *et al.*, 2009 ▶; Kuzmin *et al.*, 2013 ▶).

## 3. XANES spectroscopy of nanomaterials   

### 3.1. XANES and stoichiometry: the case of Fe_3_O_4_ nano­particles   

Magnetic NPs are nowadays the subject of extensive research because of their promising applications in many technological areas (Tartaj, 2006 ▶). The possibility of using NPs as nanodiagnostic or nanotherapeutic tools means they attract great interest in the biomedical field (Berry & Curtis, 2003 ▶). In particular, iron oxide, in the form of magnetite (Fe_3_O_4_) and/or maghemite (γ-Fe_2_O_3_), has been revealed as an ideal material because of its biocompatibility and easy removal from the body after use following natural routes (Gutiérrez *et al.*, 2006 ▶). The structural and physicochemical properties of the NPs determine to a great extent their biofunctionality, since these properties are associated with the average particle size, size distribution, shape, crystal order, surface properties and the presence of bonded molecules at the surface. Similarly, their magnetic properties depend on different parameters, such as the degree of structural order at the surface and inside the particles (Morales *et al.*, 1999 ▶). Consequently, obtaining a precise characterization of these structural details in monodisperse nanocrystals is a fundamental step to obtaining an accurate relationship with their magnetic properties.

This is of particular relevance in the case of monodisperse iron oxide nanocrystals. The great deal of information accumulated to date on the characterization of magnetite and maghemite NPs does not clarify whether the synthesized materials are single- or multiphase systems involving bulk-like oxides (Fe_3_O_4_, γ-Fe_2_O_3_, α-Fe_2_O_3_,…), or multiphase entities formed by non-stoichiometric oxides (Demortiere *et al.*, 2011 ▶). In addition, there are some indications that magnetite and maghemite NPs may show structural disorder that can substantially modify the properties of the materials and, consequently, they cannot simply be considered as small pieces of bulk material. Moreover, the existence of several polymorphs and their possible transformation through oxidation further complicates this problem.

**Figure 10:**
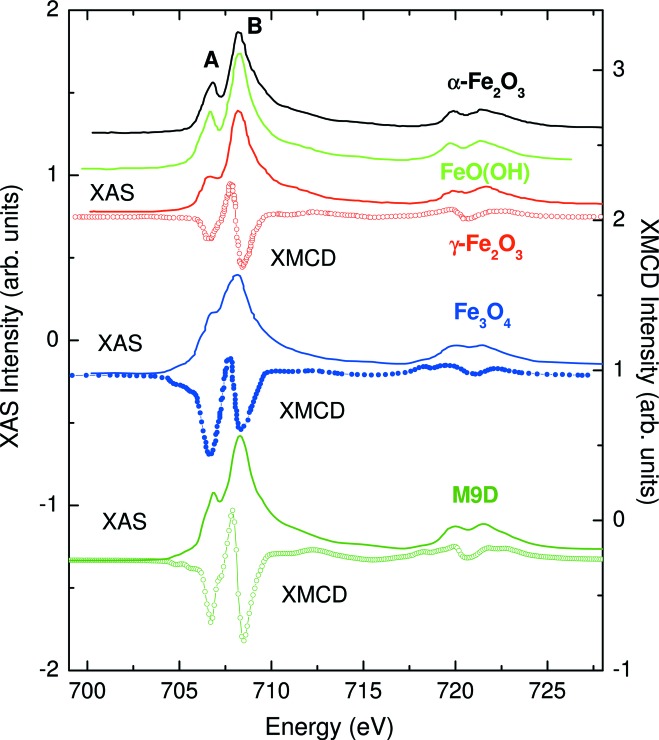
Comparison of the Fe *L*_2,3_-edges XAS and XMCD spectra of reference bulk samples [adapted from Kim *et al.* (2009 ▶) and Chang *et al.* (2012 ▶), respectively] and those of 9 nm Fe_3_O_4_ NPs (M9D) obtained by thermal decomposition [from Piquer *et al.* (2014 ▶)].

These uncertainties make clear that the standard methods currently available for solving the atomic structure of bulk crystals fail in this case, and more powerful experimental tools are needed to obtain an accurate structural and magnetic characterization of these iron oxide NPs. In this way, XAS (Sayers & Bunker, 1988 ▶; Bunker, 2010 ▶) and X-ray magnetic circular dichroism (XMCD) (van der Laan *et al.*, 1986 ▶; Schütz *et al.*, 1987 ▶; Stöhr, 1999 ▶; Funk *et al.*, 2005 ▶; Chen *et al.*, 1993 ▶; Chaboy, García, Bartolomé, Marcelli *et al.*, 1998 ▶; Chaboy, García, Bartolomé, Maruyama *et al.*, 1998 ▶; Laguna-Marco *et al.*, 2005 ▶, 2009 ▶) tools have received great attention in recent years. In principle, the study of the Fe *L*_2,3_-edge XAS spectra in Fe oxides may provide information about the oxidation state and, in some cases, allow one to separate the contributions of the magnetic moments of Fe ions in tetrahedral and octahedral sites (van Aken & Liebscher, 2002 ▶; van der Laan & Kirkman, 1992 ▶; Crocombette *et al.*, 1995 ▶). More recently, Fe *L*_2,3_-edge XMCD has also been at the centre of many of the characterization studies of nominal magnetite NPs (Park *et al.*, 2004 ▶; Goering *et al.*, 2006 ▶; Huang *et al.*, 2004 ▶; Yamasaki *et al.*, 2009 ▶). However, the most common iron oxides exhibit similar Fe *L*_2,3_ XAS and XMCD spectra because they are mainly governed by the point symmetry at the absorbing site (octahedral and tetrahedral in this case). This is illustrated in Fig. 10 ▶, where the XAS and XMCD spectra of a reference bulk material and of 9 nm Fe_3_O_4_ NPs are compared (Piquer *et al.*, 2014 ▶).

Recent XMCD studies suggest the simultaneous presence of both magnetite and maghemite in nominally magnetite NPs (Pérez *et al.*, 2009 ▶), indicating the difficulty of obtaining pure stoichiometric magnetite for particle sizes below a few tens of nanometres. Indeed, several authors suggested that magnetite and maghemite form a mixture of the form (γ-Fe_2_O_3_)_1−*x*_(Fe_3_O_4_)_*x*_, in such a way that maghemite is the dominant phase of the small 5 nm iron oxide nanocrystals, whereas the proportion of the magnetite component increases gradually as the particle size does (Park *et al.*, 2004 ▶). Unfortunately, the intrinsic limitations of Fe *L*_2,3_ XAS and XMCD do not make it possible to determine whether this mixture corresponds to a multiphase Fe_3_O_4_ + γ-Fe_2_O_3_ system, in which each component retains its bulk properties; a core–shell arrangement consisting of a magnetite core while the surface is partially oxidized (Demortiere *et al.*, 2011 ▶; Santoyo Salazar *et al.*, 2011 ▶); or a non-stoichiometric phase whose magnetic properties cannot be simply derived from the values of bulk Fe_3_O_4_ and/or γ-Fe_2_O_3_ (Piquer *et al.*, 2014 ▶).

This uncertainty in phase determination prevents one from establishing a definitive correlation between the magnetic properties and nanostructure details of iron oxide NPs and, consequently, a different approach is needed to determine the structure of Fe oxide NPs. Fe *K*-edge XAS can meet this objective because the Fe *K*-edge spectral shape is more sensitive to the geometric details of the absorbing site (overall symmetry, distances and bond angles) than the Fe *L*_2,3_-edge absorption (Wilke *et al.*, 2001 ▶; O’Day *et al.*, 2004 ▶; Berry *et al.*, 2010 ▶; Corrias *et al.*, 2000 ▶). In addition, the chemical shift associated with the change in Fe ion oxidation state and site geometry for Fe^3+^ and Fe^2+^ species is in the range ∼3–4 eV, which is easily detected experimentally (Waychunas *et al.*, 1983 ▶; Benfatto *et al.*, 2002 ▶; Okudera *et al.*, 2012 ▶; Espinosa *et al.*, 2012 ▶). Moreover, while the Fe *L*_2,3_ absorption probes only the surface of the NPs [the probing depth in total electron yield (TEY) detection mode is ∼45 Å in Fe oxides; Gota *et al.*, 2000 ▶], the Fe *K*-edge absorption probes the whole nanoparticle (Pellegrin *et al.*, 1999 ▶) and, consequently, it is possible to determine the relative amounts of the different oxide phases, if present, in the material.

**Figure 11:**
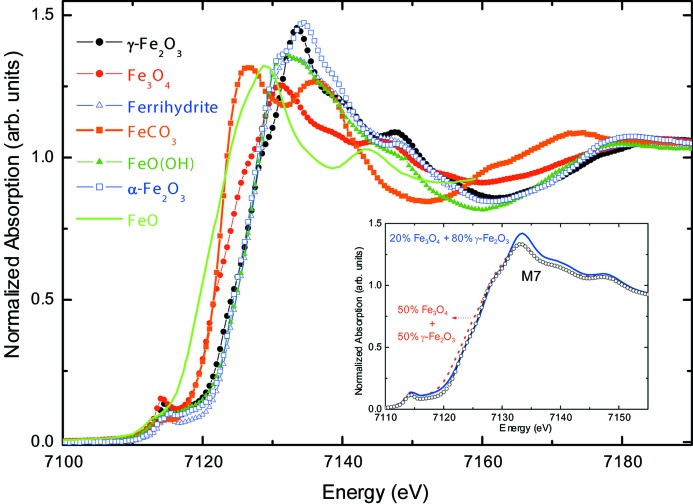
Comparison of the experimental Fe *K*-edge XANES spectra of several iron oxide bulk reference samples [from Piquer *et al.* (2013 ▶)]. In the inset, the experimental XANES spectrum of 7 nm Fe_3_O_4_ NPs (M7) is compared with the weighted addition of the spectra of bulk magnetite and maghemite reference samples.

Fig. 11 ▶ shows the Fe *K*-edge XANES spectra of several iron oxide bulk reference samples. In contrast with the *L*_2,3_-edge case, the spectral shape is clearly different for all the reference samples considered, reflecting the sensitivity of the Fe *K*-edge absorption to the geometric details of the absorbing site. In particular, magnetite and maghemite show quite different XANES spectra, not only regarding the overall spectral shape but also concerning the edge position, reflecting the different Fe—O bond lengths (Bunker, 2010 ▶; Chaboy, 2009 ▶). In a recent paper (Piquer *et al.*, 2014 ▶), an Fe *K*-edge XANES study was reported for a series of nominally Fe_3_O_4_ NPs obtained by different synthesis methods, with sizes in the range 4–30 nm. It was found that, irrespective of the sample synthesis method, none of the studied NPs displayed the characteristic XANES profile of bulk magnetite, and neither can the experimental spectra be accounted for as a linear combination of bulk oxide reference samples (Piquer *et al.*, 2014 ▶). This is illustrated in the case of 7 nm NPs in the inset of Fig. 11 ▶, showing the impossibility of simultaneously reproducing both the edge position and the white line (shape and position).

This result suggests the inadequacy of considering NPs as bulk-based materials, *i.e.* that stoichiometric bulk-like oxides can grow and coexist separately in the confined space of an NP. Moreover, the fact that the XANES spectra remain basically invariable for NPs of different sizes (Piquer *et al.*, 2014 ▶) implies that the local structural arrangement of Fe does not depend on the NP size, as expected for a core/shell scheme, but supports the existence of a single non-stoichiometric phase where the formed Fe oxide grows in a single-crystal structure with a cell parameter lying in between those of the pure stoichiometric magnetite and maghemite oxides. If this were the case then not only the structural arrangements at the Fe sites but also the distribution of the Fe^2+^ and Fe^3+^ ions, including vacancies, would depart slightly from those of bulk Fe_3_O_4_ and γ-Fe_2_O_3_ stoichiometric iron oxides. With the aim of verifying this, Piquer and co-workers performed an *ab initio* calculation of the Fe *K*-edge XANES spectra for Fe_3_O_4_ and γ-Fe_2_O_3_ clusters in which the cell parameter had been progressively modified to reach 8.364 Å, *i.e.* the average of those of the pure magnetite and maghemite compounds (Piquer *et al.*, 2014 ▶).

The modification of the cell parameter leads to an overall shift of the spectra without changing the spectral shape. When decreasing the cell parameter of Fe_3_O_4_, *i.e.* the Fe—O interatomic distance, the main absorption peak shifts to higher energies, while the opposite occurs for γ-Fe_2_O_3_ in which the cell parameter is being increased. As a result, when the computation is performed for both magnetite and maghemite clusters by imposing the same (averaged) cell parameter, the main peak lies in the middle between those of the unmodified clusters, resembling the experimental findings (Piquer *et al.*, 2014 ▶). The good agreement between the experimental and theoretical spectra strongly supports the theory that Fe oxide grows in NPs as a single spinel structure, the structural parameters and Fe^2+^/Fe^3+^ ratio of which differ from those of bulk magnetite and maghemite.

**Figure 12:**
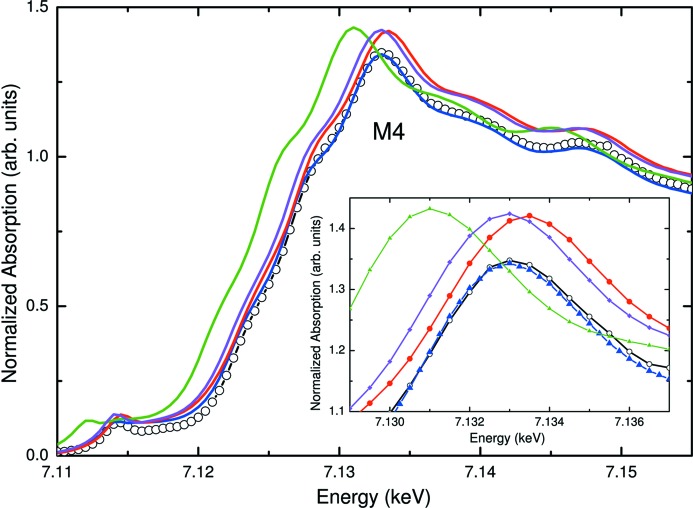
Comparison of the Fe *K*-edge XANES spectra [from Piquer *et al.* (2014 ▶)] of a 4 nm (M4) magnetite sample (black, open circles) and the weighted sums of the displaced reference samples (see text for details): 20% Fe_3_O_4_ + 80% γ-Fe_2_O_3_ (red, solid circles), 20% Fe_3_O_4_ (Δ*E* = 1 eV) + 80% γ-Fe_2_O_3_ (Δ*E* = −1 eV) (blue, solid triangles), 20% Fe_3_O_4_ + 80% γ-Fe_2_O_3_ (Δ*E* = −2.5 eV) (green, solid triangles) and 20% Fe_3_O_4_ + 80% γ-Fe_2_O_3_ (Δ*E* = −0.5 eV) (magenta, filled diamonds). The inset shows a detailed view of the white-line region.

Taking advantage of the results of the theoretical computations, a similar procedure has been applied to the experimental spectra by weighting the XANES spectra of the bulk magnetite and maghemite reference samples but shifting their energy scale prior to addition, *i.e.* simulating to some extent their expected variation upon modification of the structural parameters. (Because the Fe^2+^/Fe^3+^ ratio and vacancy distribution are unknown *a priori*, the calculations were performed by modifying the cell parameter of both magnetite and maghemite to take into account the different structural environment associated with both Fe^2+^ and Fe^3+^ ions in the spinel structure.) A comparison with the experimental spectra, reported in Fig. 12 ▶, shows excellent agreement between the experimental and calculated spectra. In the case of the smaller samples (ϕ < 9 nm), the best fit is obtained by applying Δ*E* = 1.5 eV for the magnetite reference spectrum and Δ*E* = −0.5 eV for the maghemite one, which agrees with the fact that the experimental edge position (and consequently the bond length) is closer to maghemite, whereas in the case of the larger samples (ϕ ≥ 9 nm) good agreement is obtained by a 50% weighting of the Fe_3_O_4_ and γ-Fe_2_O_3_ after shifting them by Δ*E* = ±0.75 or ±1 eV, *i.e.* intermediate between those of the magnetite and maghemite bulk reference samples.

All in all, these results indicate that the Fe *L*_2,3_-edge absorption alone is not able to determine the structure of nanosized magnetite NPs. While the Fe *L*_2,3_-edge absorption is dictated by the valence and point symmetry of the absorbing sites, the overall line shape of all the iron oxides is similar, making it complicated to identify the relative amounts of the oxide phases present in the material. In contrast, the Fe *K*-edge spectral shape is clearly different for all the reference samples considered, reflecting the high sensitivity of the Fe *K*-edge absorption to the geometric details of the absorbing site.

Study of the XAS data recorded on a series of nominally Fe_3_O_4_ NPs with sizes in the range 4–30 nm indicates that none of the synthesized NPs can be identified as a mixture of stoichiometric bulk-like iron oxides, independently of the sample synthesis method. The experimental spectra are not well reproduced by any linear combination of the absorption spectra of Fe_3_O_4_ and γ-Fe_2_O_3_ bulk reference samples, even taking into account other oxides such as goethite or ferri­hydrite. The failure of this hypothesis reflects the inadequacy of considering NPs as bulk-based materials, *i.e.* that stoichiometric bulk-like oxides can grow and coexist separately in the confined space of an NP. Moreover, analysis of the Fe *K*-edge spectra indicates that the local structural arrangement of Fe does not depend on the NP size as expected for a core/shell scheme. On the contrary, these results indicate that the synthesis of Fe_3_O_4_ magnetite NPs leads to the growth of a single-phase non-stoichiometric oxide, the crystal structure of which possesses a cell parameter lying in between those of the pure stoichiometric magnetite and maghemite oxides. All these results suggest that a single phase develops inside the NPs during the synthesis process and that its structural details are mainly determined by steric effects; that is, the partial oxidation of the nominal magnetite NPs comes mainly from a greater disorder in the octahedral sub-network, allowing the appearance of vacancies at the spinel octahedral sites. This disorder, caused by size constraints, leads to the modification of the structural arrangements at the Fe sites with respect to those found in bulk-like iron oxides. This new intermediate phase can be seen as a mixture of ‘structurally adapted’ magnetite and maghemite oxides, in such a way that magnetite and maghemite rearrange their crystallographic structures in order to obtain the same crystal cell parameter. It is thus the size of the NPs which determines the variation in the cell parameter and the distribution of vacancies.

### 3.2. The role of vacancies in XANES: the case of ZnO-based DMSs   

The second example concerns the detection of vacancies using XANES. We shall discuss this in connection with the development of oxide-based dilute magnetic semiconductors (DMS), *i.e.* semiconductors containing a small amount of magnetic impurities and showing high-temperature ferromagnetism (HTFM) (Ohno, 1998 ▶; Dietl *et al.*, 2000 ▶; Sato & Katayama-Yoshida, 2001 ▶; Matsumoto *et al.*, 2001 ▶). The origin of this HTFM remains controversial and several authors have proposed that it is caused by defects such as oxygen and zinc vacancies, as well as hydrogen, oxygen and zinc interstitials (Sanchez *et al.*, 2008 ▶; Gallego *et al.*, 2005 ▶; Liu & Jiang, 2010 ▶; Hong *et al.*, 2007 ▶; Wang *et al.*, 2008 ▶; Patterson, 2006 ▶). In this respect, XAS has been applied with the aim of determining the exact type of defect that causes HTFM in these systems. However, based on similar experimental spectra, different authors report opposing conclusions regarding the nature of defects involved in the observed HTFM. Hsu *et al.* (2006 ▶) have concluded that oxygen vacancies enhance room-temperature ferromagnetism in Co-doped ZnO films while, on the contrary, Yan *et al.* (2007 ▶) concluded that Zn vacancies induce room-temperature ferromagnetism in Mn-doped ZnO. This scenario is further complicated by the results of Zhang *et al.* (2010 ▶), who concluded from similar data that the aforesaid oxygen vacancies are located in the second shell around the magnetic ions.

The fact that the analysis of similar experimental spectra leads to opposing conclusions (Hsu *et al.*, 2006 ▶; Yan *et al.*, 2007 ▶; Zhang *et al.*, 2010 ▶) poses a question about the reliability of these assignments, as well as of the capability of XAS itself to determine the presence of vacancies in the materials under study and, consequently, to shed light on the origin of magnetism in these systems. Indeed, the determination of the presence of vacancies from XANES spectra is not an easy task. It should be noted that the conclusions reported previously were derived from the occurrence of subtle changes in the intensity of different spectral features through fingerprint comparisons of *ab initio* computations and experimental data. Consequently, a convincing demonstration of the influence of vacancies on XANES spectra, beyond qualitative fingerprint analysis, is still missing.

**Figure 13:**
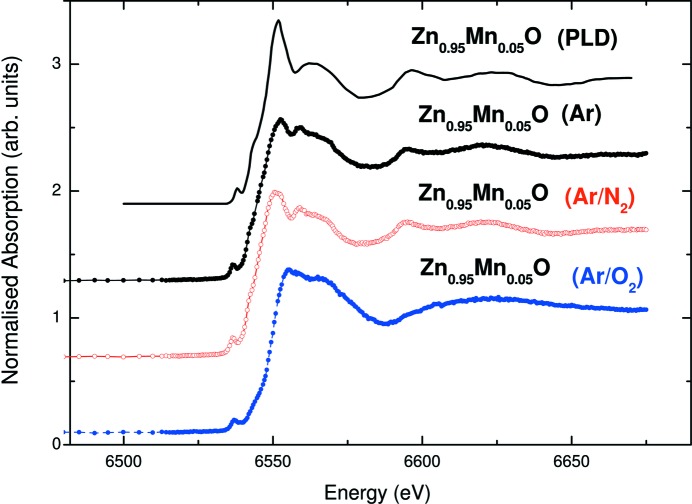
Comparison of experimental Mn *K*-edge XANES spectra [from Guglieri *et al.* (2011 ▶)] of Ar-, Ar/N_2_- and Ar/O_2_-prepared Zn_0.95_Mn_0.05_O films and experimental data (PLD) reported by Pellicer-Porres *et al.* (2006 ▶) for Mn in ZnO wurtzite (w) structures.

With this in mind, Guglieri *et al.* (2011 ▶) performed a systematic *ab initio* computation of both the Mn and Zn *K*-edge XANES spectra in Zn_0.95_Mn_0.05_O films showing HTFM behaviour. The Mn *K*-edge spectra of Zn_0.95_Mn_0.05_O films prepared with different sputtering gases are shown in Fig. 13 ▶, being similar to those previously reported from which several authors concluded the existence of either oxygen or zinc vacancies (Hsu *et al.*, 2006 ▶; Yan *et al.*, 2007 ▶; Zhang *et al.*, 2010 ▶). However, the near-edge region of the spectra varies with the sputtering gas used, as do their magnetic properties (Céspedes *et al.*, 2010 ▶; Céspedes, 2009 ▶). Therefore, Guglieri and co-workers explored whether these differences can be assigned to the presence of vacancies or, on the contrary, if they are associated with different Mn short-range structural order in the lattice, as was proposed in similar ZnMnO systems (Céspedes *et al.*, 2007 ▶, 2008 ▶).

To this end, several theoretical calculations were performed by imposing the presence of both oxygen and zinc vacancies in the first coordination shells of the photoabsorbing atom (Guglieri *et al.*, 2011 ▶). The computations were carried out using the multiple-scattering program *CONTINUUM* (Natoli *et al.*, 1980 ▶), included in the *MXAN* program package (Benfatto & Della Longa, 2001 ▶). A complete discussion of the procedure can be found elsewhere (Chaboy & Quartieri, 1995 ▶; Chaboy, 2009 ▶; Chaboy *et al.*, 2005 ▶). The calculations have been done, at both the Zn *K*-edge and the Mn *K*-edge, for a ZnO cluster including the contributions for atoms within the first 8 Å around the photoabsorber. In all cases, the interatomic distances were kept fixed as in the undoped wurtzite ZnO (w-ZnO). The Mn *K*-edge spectra were calculated by simply substituting Mn with Zn at the photoabsorbing site (Guglieri *et al.*, 2011 ▶).

**Figure 14:**
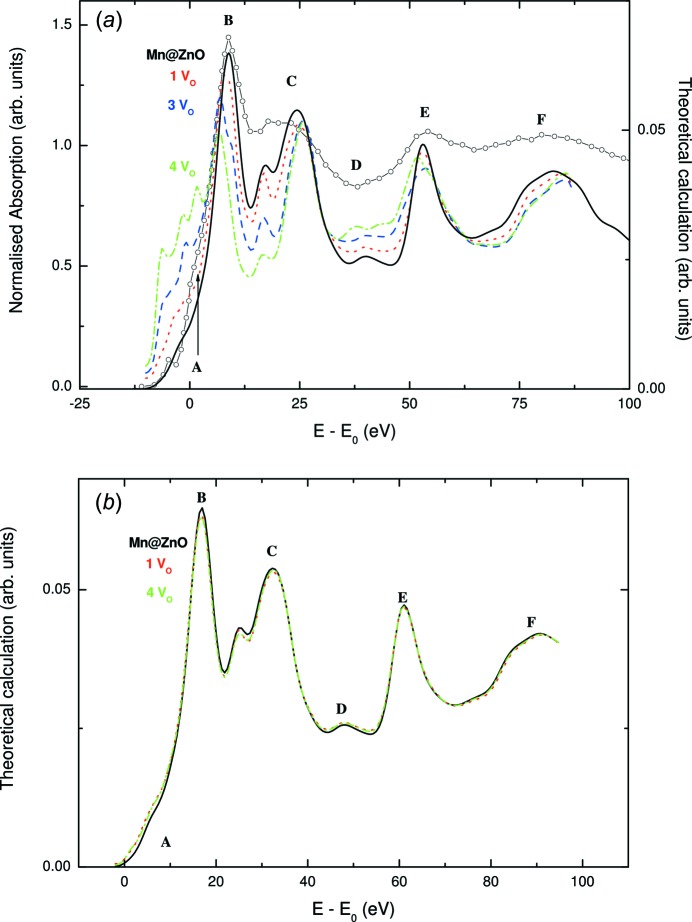
(*a*) A comparison of the Mn *K*-edge XANES spectrum of wurtzite-type w-Mn:ZnO (open circles) [from Guglieri *et al.* (2011 ▶)] and the theoretical calculations performed by considering the existence of oxygen vacancies (*V*_O_) in the first coordination shell of the photoabsorbing atoms: no vacancies (black, solid line), 1 *V*_O_ (red, dotted line), 3 *V*_O_ (blue, dashed line) and 4 *V*_O_ (green, dot-dashed line). (*b*) The same as part (*a*) but considering a binomial distribution of 50% of oxygen vacancies (see text for details).

As shown in Fig. 14 ▶, the presence of a single oxygen vacancy in the tetrahedron surrounding Zn(Mn) affects the whole spectral shape, *i.e.* the relative intensity of all the spectral features is modified. This is the expected result (Kuzmin *et al.*, 2007 ▶), contrary to previous reports (Hsu *et al.*, 2006 ▶), because the oxygen vacancy affects not only the single scattering process in the first coordination shell of Zn but also many of the multiple scattering paths contributing to XANES. Including further oxygen vacancies in the next-neighbouring tetrahedron enhances these differences, whereas the inclusion of Zn vacancies in the second coordination shell does not significantly modify the spectral shape (Guglieri *et al.*, 2011 ▶).

It has previously been reported that, upon removing one O atom from the first coordination shell, a new peak appears in the pre-edge region, and its intensity becomes more pronounced if a second oxygen vacancy is created in the second oxygen coordination shell (Hsu *et al.*, 2006 ▶). Beyond the lack of agreement with the calculations above, this model is not reliable as it considers an *ad hoc* arrangement of vacancies. If the oxygen vacancies are randomly distributed, the probability of photoabsorbing Zn(Mn) having *n* vacancies in the first coordination shell can be calculated by a simple binomial distribution as 
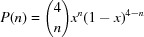
, where *x* is the concentration of oxygen vacancies. Accordingly, the Zn(Mn) *K*-edge XANES spectrum should correspond to the addition of spectra with and without oxygen vacancies around the photoabsorbing Zn atom, weighted according to this probability. As shown in Fig. 14 ▶ in the case of the Mn *K*-edge, the effects induced by the presence of oxygen vacancies are undetectable for defect concentrations of up to 50%.

These results indicate that the existence of both oxygen and Zn vacancies in Mn:ZnO films has little influence on the absorption spectra recorded at either the Zn or Mn *K*-edge. Indeed, the theoretical computations presented here indicate that, if a reliable concentration of defects, randomly distributed, is taken into account, the effect of vacancies on the XANES spectra is negligible. Consequently, these results suggest that the disagreement between the theoretical computations and the Mn *K*-edge of the Zn_0.95_Mn_0.05_O thin film in the wurtzite structure is not due to the presence of vacancies. In this situation, structural modifications induced by the Mn substitution at the Zn site appear to be the best explanation to account for the disagreement between the theoretical and experimental spectra (Céspedes, 2009 ▶; Smolentsev *et al.*, 2007 ▶).

**Figure 15:**
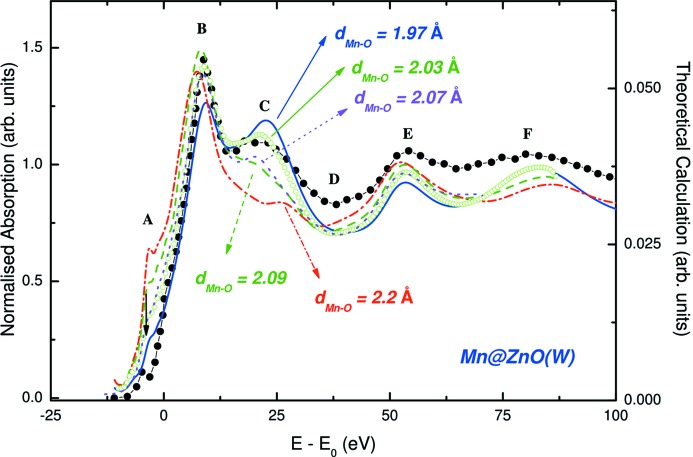
A comparison between the experimental Mn *K*-edge XANES of Mn:ZnO (filled circles) [from Guglieri *et al.* (2011 ▶)] and computations performed by considering that Mn substitutes for Zn in the wurtzite ZnO structure, adapting the interatomic Mn—O distance in the first coordination shell.

The computations above were performed by considering that Mn substitutes for Zn in the w-ZnO structure without modifying the interatomic distances. In this structure the nearest-neighbour interatomic distance is *R*_Zn—O_ = 1.97 Å, while *R*_Mn—O_ = 2.22 Å in MnO. Therefore, it seems reasonable to think that, on entering the w-ZnO structure, Mn adapts the original ZnO_4_ tetrahedron by enlarging the *R*_Mn—O_ distances. To verify this hypothesis, Guglieri *et al.* calculated the Mn *K*-edge XANES spectrum of Mn:ZnO in the wurtzite phase by considering that Mn substitutes for Zn in the w-ZnO framework and, in addition, by progressively increasing the interatomic *R*_Mn—O_ distance in the nearest-neighbour shell of Mn (MnO_4_ tetrahedron) from 1.97 Å, w-ZnO-like, to 2.22 Å, as in MnO. As shown in Fig. 15 ▶, the computation performed considering *R*_Mn—O_ = 2.03 Å yields a good reproduction of the experimental spectrum, especially regarding the broad resonance *C*, ∼20 eV above the edge, and the intensity ratio between this resonance and the white-line (peak *B*). Moreover, the computation exhibits a shift towards lower energies of the edge position, as expected because *R*_Mn—O_ increases (Chaboy, 2009 ▶), and the structure at the rising edge is enhanced slightly with respect to the computation in which Mn simply substitutes for Zn without modifying the interatomic distances. Finally, it was also checked in this case that adding an oxygen vacancy in the first coordination shell of Mn has no effect on the calculated spectrum. This behaviour is in agreement with the modification observed in the XANES spectrum of the Zn_0.95_Mn_0.05_O thin films prepared using different sputtering gases (see Fig. 13 ▶). These results are in agreement with those of Smolentsev *et al.* (2009 ▶) which showed that slight changes in the structural parameters of Mn:ZnO films have a profound influence on the characteristics of photoelectron scattering in the vicinity of Mn. This indicates that the variation in the reported HTFM in Ar–Zn_0.95_Mn_0.05_O and Ar/N_2_–Zn_0.95_Mn_0.05_O samples, where the ferromagnetic response is diminished when using Ar/N_2_, is mainly associated with dissimilar Mn local structures in the two samples.

Concluding, the XANES results obtained in the study of Zn_0.95_Mn_0.05_O thin films prepared with different sputtering gases and presenting room-temperature ferromagnetic behaviour indicate, on the one hand, that Mn substitutes for Zn in the wurtzite structure. However, contrary to previous claims, the results of comparisons between the experimental and theoretical spectra indicate that, by assuming a reliable defect concentration randomly distributed, the presence of neither oxygen nor zinc vacancies is detectable in the XANES spectra. Indeed, the theoretical computations presented here indicate that Mn adapts its local environment by increasing the Mn—O interatomic distance with the nearest-neighbour oxygen atoms. This modification is slightly different for Zn_0.95_Mn_0.05_O thin films prepared with different sputtering gases, which suggests that the different magnetic behaviour observed for the different samples is related to the different local structure of Mn in the films.

### 3.3. XANES and charge-transfer effects: the case of ZnO nanoparticles   

Finally, we wish to discuss the possibility of using XANES to detect charge-transfer effects at the surface and/or interface of new tailored nanomaterials. Very often, experimental support for these effects is derived from modifications to the XANES spectra. In this way, early work by García *et al.* (2007 ▶) reported the occurrence of room-temperature ferromagnetism in ZnO NPs capped with different organic molecules. The study of the Zn *K*-edge XANES spectra showed modification of the near-edge region and, in particular, of the white-line (see peak *B* in Fig. 15 ▶) intensity as a function of the organic molecule used. The strongest modification of the spectral profile of bulk ZnO was found in the case of NPs capped with dodecanethiol, *i.e.* when the molecule bonds to the particle surface through an S atom. These results were interpreted as reflecting the alteration of the electronic configuration of the ZnO system due to charge-transfer effects between Zn and S, making a parallel with the charge-transfer model used to account for the peculiar magnetic behaviour of thiol-capped Au NPs (Crespo *et al.*, 2004 ▶; Yamamoto *et al.*, 2004 ▶). Similarly, Chen *et al.* (2012 ▶) have interpreted the observed modifications of the O *K*-edge XANES spectra of Al-doped ZnO NPs as being due to charge-transfer effects of Zn and Al.

However, a common characteristic of the works proposing the existence of charge-transfer effects in these ZnO-based systems from the analysis of XANES is the fact that they do not consider at all the influence of possible structural modifications due to the capping or doping of the ZnO NPs. Contrary to the case of the huge white lines found in the *L*_2,3_-edges of lanthanides and 5*d* metals associated with the well localized 5*d* states (Qi *et al.*, 1987 ▶; Chaboy *et al.*, 1995 ▶), the delocalized character of the final *p*-states probed in *K*-edge absorption prevents the establishment of a direct relationship between spectroscopic intensity and the density of states and/or hole-count changes (Laguna-Marco *et al.*, 2008 ▶; Chaboy *et al.*, 2007 ▶). In addition, the XANES region of the absorption spectrum is highly sensitive to the bonding geometry and the details of the white line are also determined by the interatomic distances (the shorter the distance the broader the white line) (Bunker, 2010 ▶). Consequently, special attention will be paid to identify the variation in white-line intensity with charge-transfer effects.

**Figure 16:**
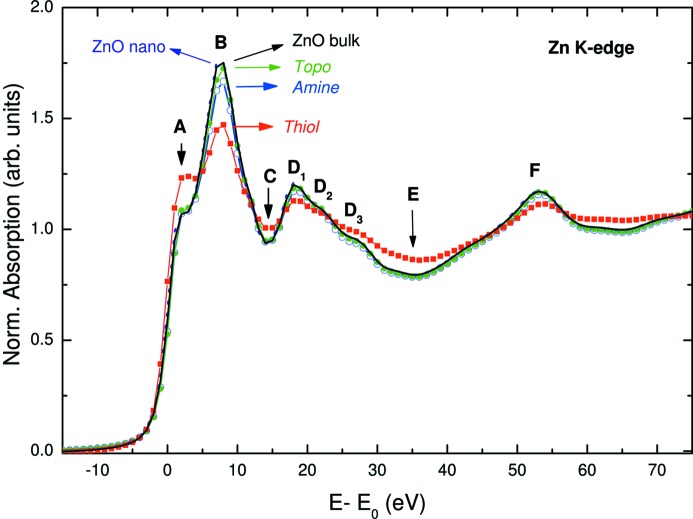
A comparison of the experimental Zn *K*-edge XANES spectra for ZnO nanoparticles capped with Topo (green, filled circles), Amine (blue, open circles) and Thiol (red, filled squares) [from Guglieri & Chaboy (2010) ▶ and Guglieri *et al.* (2012 ▶)]. For the sake of comparison, the XANES spectra of both bulk ZnO (black, solid line) and ZnO nanopowder (purple, dotted line) are also shown.

To illustrate this we present in the following the Zn *K*-edge XANES study of ∼20 nm ZnO NPs capped with three different organic molecules: tryoctylphosphine (Topo), dodecylamine (Amine), and dodecanethiol (Thiol), which bond to the particle surface through an O, N and S atom, respectively. As shown in Fig. 16 ▶, in the case of the uncapped ZnO samples the Zn *K*-edge spectrum is characterized by a main absorption peak (*B*) and a positive spectral feature (*D*_1_) with a double shoulder-like structure at higher energies (*D*_2_, *D*_3_). This spectroscopic profile is retained in the case of ZnO NPs capped with Amine and Topo. In contrast, the intensity of the main absorption line of the ZnO sample capped with Thiol shows a strong reduction, as previously reported (García *et al.*, 2007 ▶). However, not only the white-line intensity but also the overall spectral shape in the near-edge region are strongly modified with respect to bulk ZnO. In particular, the intensity of the low-energy shoulder (*A*) of the main absorption line grows and, at the same time, an overall reduction in amplitude is observed. This behaviour suggests that the observed variations in the XANES spectrum are associated with structural effects rather than exclusively with charge-transfer effects.

Taking into account that the Zn—S interatomic distance in ZnS is ∼2.34 Å, while the Zn—O distance in ZnO is 1.97 Å, it seems reasonable to think that, upon formation of Zn—S bonds at the surface of the NPs, the spectral shape and the intensity of the main XANES features might change, even in the absence of charge-transfer effects. Hence, a detailed *ab initio* computation of the Zn *K*-edge XANES spectra of these ZnO NPs was performed by considering a different local structure of the Zn atoms in the inner part of the NP and those at the surface, where the bonding with the capping molecule takes place (Chaboy *et al.*, 2010 ▶; Guglieri & Chaboy, 2010 ▶).

**Figure 17:**
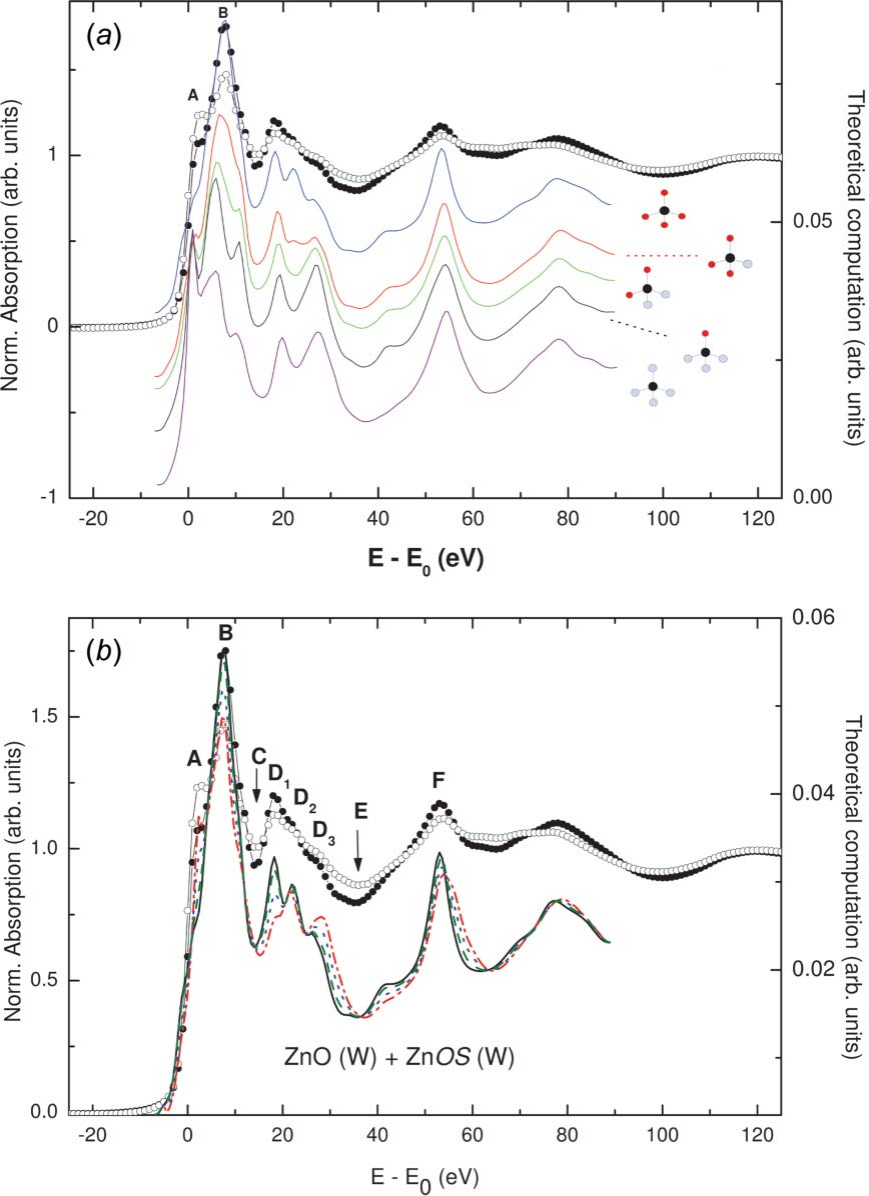
(*a*) A comparison of the experimental Zn *K*-edge XANES spectra of bulk ZnO (filled circles) and the Thiol sample (black, open circles) [from Guglieri *et al.* (2014 ▶)] and the theoretical signal computed for a ZnO cluster in which the oxygen atoms in the first coordination shell have been progressively substituted by S atoms and the Zn—S interatomic distance is set equal to that of bulk ZnS. (*b*) The same as part (*a*) for the case of spectra obtained by adding the theoretical signal computed for an 8 Å ZnO cluster and the same cluster in which the four O next-neighbours have been substituted by S and by imposing the Zn—S interatomic distance of w-ZnS (ZnOS) with different relative weights: pure ZnO (black, solid line), 90% ZnO + 10% ZnOS (green, dashed line), 75% ZnO + 25% ZnOS (blue, dotted line), 50% ZnO + 50% ZnOS (red, dot-dashed line).

In order to assess the aforesaid possibility, the computations were performed for a ZnO cluster including coordination shells within the first 8 Å around ZnO, *i.e.* as expected for bulk-like ZnO XANES, and for the same cluster but progressively substituting the four next-neighbour O atoms with S ones. This substitution was made at the same crystallographic positions, *i.e.* assuming a Zn—S interatomic distance equal to the Zn—O one, and also by increasing the Zn—S bond length as for wurtzite-ZnS (w-ZnS). As shown in Fig. 17 ▶, despite the fact that only four atoms have been changed in the cluster of 177 atoms, the effect on the XANES spectrum is dramatic, especially concerning the near-edge region. Moreover, it is observed that, while the simple substitution of S at the oxygen positions does not reproduce the experimental spectrum, when the interatomic distance of the substituted Zn—S bonds is set equal to that of w-ZnS (hereinafter ZnO—S), the theoretical spectrum resembles the experimental Thiol one. In particular, the intensity ratio of peaks *A* and *B* is inverted in both ZnO and ZnO—S calculations, which mimics the experimental differences of the Zn *K*-edge XANES spectra of both bulk ZnO and Thiol-capped ZnO NPs samples: the intensity of the main absorption line (*B* peak) decreases and that of the low-energy *A* peak increases as the ZnO NPs are capped with Thiol. Finally, the expected Zn *K*-edge XANES signal was built up by considering that the experimental XANES spectrum corresponds to the weighted addition of contributions from Zn atoms within the ZnO frame at the inner part of the particle and from those bonded to S near the surface. The results, reported in Fig. 17 ▶, show that, as the weight of the ZnS contribution increases, the computed signal agrees with the observed evolution of XANES from bulk ZnO to the Thiol sample.

These results indicate, on the one hand, that the observed modifications of the Zn *K*-edge XANES spectra in these capped ZnO NPs are due to structural modifications and not to charge-transfer effects. The case of samples capped with Thiol is especially significant as the XANES spectrum is highly affected by the Zn—S scattering contributions, and comparison of the experimental spectra and *ab initio* computations indicates the formation of a well defined ZnS interface at the surface of the nanoparticle in which ZnS adopts the local structure of wurtzite. Therefore, these findings suggest that the HTFM behaviour observed in these NPs is related to this interface, the details of which (thickness, interpenetration, *etc.*) should determine the particular magnetic properties of each system (Guglieri *et al.*, 2012 ▶, 2013 ▶).

A similar analysis can be applied to the study of the O *K*-edge in Al-doped ZnO (AZO) NPs and films showing HTFM behaviour (Ma *et al.*, 2009 ▶; Gao *et al.*, 2010 ▶; Xing *et al.*, 2013 ▶; Chen *et al.*, 2009 ▶). While several authors concluded that metallic clusters of Al are responsible for the observed HTFM (Ma *et al.*, 2009 ▶), it has also been proposed that this behaviour arises from charge transfer between Zn and Al (Chen *et al.*, 2012 ▶). In this scenario, in which both structural and electronic effects are claimed to be responsible for the HTFM behaviour, X-ray absorption spectroscopy (XAS) constitutes an incomparable tool due to its capability of simultaneously providing element-selective electronic and structural information.

**Figure 18:**
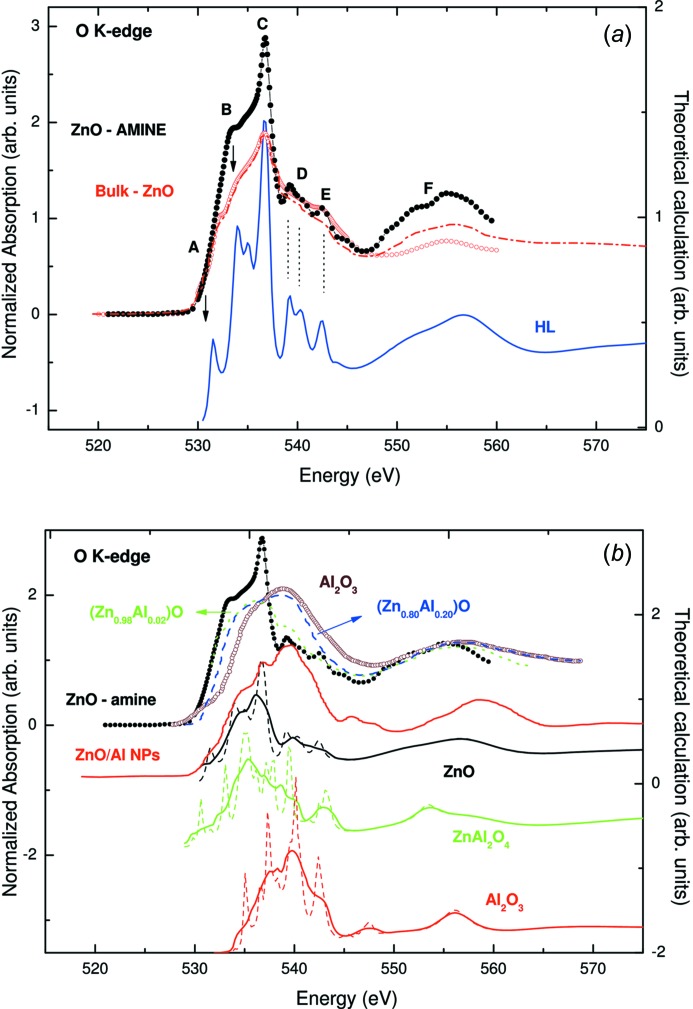
(*a*) A comparison of the room-temperature O *K*-edge XAS spectra of bulk ZnO (black solid circles) and dodecylamine-capped (Amine) ZnO NPs (red, open circles) [from Guglieri *et al.* (2014 ▶)] and the theoretical computations. For the sake of comparison, the ZnO spectrum reported by Chen *et al.* (2012 ▶) has been included (red, dot-dashed line). (*b*) A comparison of the O *K*-edge XAS spectra of Al-doped ZnO nanoparticles and films [from Chen *et al.* (2012 ▶) and Ma *et al.* (2009 ▶), respectively] and the theoretical computations performed for ZnO, ZnAl_2_O_4_ and Al_2_O_3_. The computed spectra (dashed lines) have been convoluted with a Lorentzian shape function to account for the broadening associated with the core–hole lifetime and the experimental resolution.

The normalized O *K*-edge XAS spectra recorded on Al-doped ZnO NPs [Al_0.2_/(ZnO)_0.8_] and Zn_1−*x*_Al_*x*_O films reported by, respectively, Chen *et al.* (2012 ▶) and Ma *et al.* (2009 ▶), are compared in Fig. 18 ▶. The XAS spectrum of the as-prepared Al_0.2_/(ZnO)_0.8_ NPs is similar to that of bulk ZnO, although the intensities of the main spectral features were found to decrease slightly compared with those of pure ZnO (Chen *et al.*, 2012 ▶). This reduction was assigned to a decrease in the available empty O 2*p* states and interpreted in terms of charge transfer from Al to O. The same argument was extended to the case of a sample annealed at 923 K (marked ZnO/Al NPs in Fig. 18 ▶), concluding that, because the intensity is further reduced, a greater degree of occupancy of the 2*p* states of O in the ZnO matrix takes place in the system as a consequence of increased charge transfer from adsorbed Al. In this way, the observed increase in saturation magnetization is related to the aforesaid charge transfer from adsorbed Al to ZnO surfaces, giving rise to the observed ferromagnetic behaviour in ZnO/Al NPs (Chen *et al.*, 2012 ▶). However, it is worth noting in this respect that the spectral shape of the sample subject to annealing, showing HTFM, is completely different from that of ZnO, suggesting the occurrence of a dramatic structural change in the sample. Indeed, the normalized O *K*-edge XAS spectra recorded on Al-doped ZnO NPs [Al_0.2_/(ZnO)_0.8_] and Zn_1−*x*_Al_*x*_O films show that, while the spectrum of the nonmagnetic (Zn_0.98_Al_0.02_)O film looks similar to that of pure ZnO, that of the magnetic (Zn_0.80_Al_0.20_)O film is significantly different from that of pure ZnO and its O *K*-edge is more related to that of amorphous Al_2_O_3_. Combined with photoluminescence data, these results were interpreted as the observed ferromagnetism being due to oxygen vacancies at the surface or grain boundaries (Schoenhalz *et al.*, 2009 ▶) of ZnO nanocrystals in the amorphous matrix (Ma *et al.*, 2009 ▶). As a consequence, we are faced with two opposing explanations to account for the observed modification of the XAS spectra, *i.e.* these changes are either due to electronic effects or they are due to structural changes associated with the Al–ZnO interaction.

Trying to shed light on this problem, Guglieri and Chaboy (Guglieri & Chaboy, 2014 ▶) have studied how the modification of the local structural environment of oxygen affects the spectral shape using a detailed *ab initio* calculation of the O *K*-edge XANES spectrum with the multiple-scattering program *CONTINUUM* (Natoli *et al.*, 1980 ▶). A complete discussion of the procedure can be found elsewhere (Guglieri *et al.*, 2014 ▶). The initial computations were performed for a wurtzite-like w-ZnO cluster, including contributions from neighbouring atoms located within the first 8 Å around the photoabsorbing oxygen atom. As shown in Fig. 18 ▶, this calculation leads to the correct reproduction of the shape and energy position of the different spectral features and of both their relative energy separation and the intensity ratio (Guglieri *et al.*, 2014 ▶). It should be noted that the computations were performed using neutral charge densities, *i.e.* no charge transfer has been considered. The good agreement between the experimental and theoretical spectra indicates that the local structure is the main factor governing the experimental spectral shape and, consequently, only subtle changes are expected if charge-transfer effects occur. Consequently, the dramatic changes in the XANES spectra observed for the Al-doped samples showing ferromagnetic behaviour cannot be accounted for in terms of charge-transfer effects but they do suggest the occurrence of structural effects.

With the aim of discerning the structural origin of the observed results different possibilities were explored, *i.e.* that dopant Al ions diffuse into the ZnO matrix and occupy substitutional locations (Wang *et al.*, 2013 ▶), or the formation of an interface between a ZnO nanowire core and an Al_2_O_3_ shell. The results of the calculations (not shown) clearly indicate that Al does not enter the ZnO matrix. The existence of a chemical reaction product between ZnO and Al after thermal treatment has been also considered, as proposed in XAS studies on Zn-doped Al_2_O_3_ (Wang *et al.*, 2005 ▶) or for Al-doped ZnO nanowires (Xing *et al.*, 2013 ▶). Accordingly, the computation of the O *K*-edge XAS spectrum was also performed in the case of both ZnAl_2_O_4_ and Al_2_O_3_ (Guglieri & Chaboy, 2014 ▶). In the former case the next-neighbour oxygen arrangement is formed by three Al atoms at ∼1.91 Å and one Zn at 1.95 Å, *i.e.* Zn is still present in the first coordination shell, whereas it is absent in the case of Al_2_O_3_. The results of the computations, displayed on a unique energy scale, are shown in Fig. 18 ▶. The calculations show that, in the case of ZnAl_2_O_4_, the spectral intensity in the low-energy region of the spectrum increases compared with that of w-ZnO. Starting from pure w-ZnO it is observed that, by increasing the oxygen contribution to the spectra coming from a ZnAl_2_O_4_-like arrangement, the main absorption features become broader and the intensity of the main absorption peak decreases. This is in agreement with the modification of the ZnO experimental spectrum observed for the low-content Al films. Moreover, the shift to higher energies, together with further spectroscopic broadening of the absorption peaks observed in both films and annealed NPs, *i.e.* those compounds showing ferromagnetism, resembles that of pure Al_2_O_3_. All in all, these results suggest the existence of a dramatic structural modification of the original wurtzite w-ZnO structure at the surface of the materials, leading to the formation of Al-rich regions with prevailing Al—O bonds. The observed broadening of the spectra indicates that these regions are strongly disordered. These results are in agreement with those previously found in the case of capped ZnO NPs, suggesting that HTFM is associated with the details of the formed interface.

## 4. Conclusions   

X-ray absorption spectroscopy is well suited to the investigation of nanomaterials, and size-dependent effects are readily detectable for nanoparticles having a size below about 10–15 nm. Analysis of the nearest coordination shells around a photoabsorber can be performed using conventional methods of analysis to determine the local structure parameters and degree of disorder. However, more advanced simulation techniques should be used to describe the structure and dynamics of a nanoparticle as a whole. Among these, classical molecular dynamics has some advantages, due to the small number of force-field model parameters, the ability explicitly to incorporate thermal and static disorder effects, and relatively low computational costs. At the same time, it is limited to the high-temperature region and fails when strong electron–phonon interactions occur. In this case, the reliability of the approach can be improved and extended by employing more accurate but computationally much more heavy *ab initio* molecular dynamics simulations, allowing the parameter-free analysis of experimental EXAFS data. In the case of XANES, the comparison of experimental and *ab initio* theoretical spectra demonstrates that the modification of the spectral shape associated with the change in size scale is mainly determined by local structure effects. Consequently, special attention should be paid to addressing the occurrence of effects due to the nanosized nature of the materials, such as charge-transfer effects, the existence and type of vacancies *etc.*, based only on the modification of the XANES spectra compared with the bulk ones.

## References

[bb1] Abraham, F. F. (1986). *Adv. Phys.* **35**, 1–111.

[bb2] Agostini, G., Grisenti, R., Lamberti, C., Piovano, A. & Fornasini, P. (2013). *J. Phys. Conf. Ser.* **430**, 012031.

[bb3] Agostini, G., Piovano, A., Bertinetti, L., Pellegrini, R., Leofanti, G., Groppo, E. & Lamberti, C. (2014). *J. Phys. Chem. C*, **118**, 4085–4094.

[bb4] Aken, P. A. van & Liebscher, B. (2002). *Phys. Chem. Miner.* **29**, 188–200.

[bb5] Aksenov, V., Koval’chuk, M., Kuz’min, A., Purans, Y. & Tyutyunnikov, S. (2006). *Crystallogr. Rep.* **51**, 908–935.

[bb6] Ankudinov, A. L., Ravel, B., Rehr, J. J. & Conradson, S. D. (1998). *Phys. Rev. B*, **58**, 7565–7576.

[bb7] Anspoks, A., Kalinko, A., Kalendarev, R. & Kuzmin, A. (2012). *Phys. Rev. B*, **86**, 174114.

[bb8] Anspoks, A., Kalinko, A., Kalendarev, R. & Kuzmin, A. (2014). *Thin Solid Films*, **553**, 58–62.

[bb9] Anspoks, A., Kalinko, A., Timoshenko, J. & Kuzmin, A. (2014). *Solid State Commun.* **183**, 22–26.

[bb10] Anspoks, A. & Kuzmin, A. (2011). *J. Non-Cryst. Solids*, **357**, 2604–2610.

[bb11] Anspoks, A., Kuzmin, A., Kalinko, A. & Timoshenko, J. (2010). *Solid State Commun.* **150**, 2270–2274.

[bb12] Ashley, C. A. & Doniach, S. (1975). *Phys. Rev. B*, **11**, 1279–1288.

[bb13] Avendaño, E., Kuzmin, A., Purans, J., Azens, A., Niklasson, G. A. & Granqvist, C. G. (2005). *Phys. Scr.* T**115**, 464–466.

[bb14] Babanov, Y. A., Vasin, V. V., Ageev, A. L. & Ershov, N. V. (1981). *Phys. Status Solidi B*, **105**, 747–754.

[bb15] Baker, S. H., Roy, M., Gurman, S. J. & Binns, C. (2009). *J. Phys. Condens. Matter*, **21**, 183002. 10.1088/0953-8984/21/18/183002 21825446

[bb16] Bakushinsky, A. & Goncharsky, A. (1994). *Ill-Posed Problems: Theory and Applications.* Dordrecht: Kluwer Academic Publishers.

[bb17] Benfatto, M. & Della Longa, S. (2001). *J. Synchrotron Rad.* **8**, 1087–1094. 10.1107/s0909049501006422 11486360

[bb18] Benfatto, M., Solera, J. A., García, J. & Chaboy, J. (2002). *Chem. Phys.* **282**, 441–450.

[bb19] Beni, G. & Platzman, P. M. (1976). *Phys. Rev. B*, **14**, 1514–1518.

[bb20] Berry, C. C. & Curtis, A. S. G. (2003). *J. Phys. D Appl. Phys.* **36**, R198–R206.

[bb21] Berry, A. J., Yaxley, G. M., Woodland, A. B. & Foran, G. J. (2010). *Chem. Geol.* **278**, 31–37.

[bb22] Bianchi, A. E., Plivelic, T. S., Punte, G. & Torriani, I. L. (2008). *J. Mater. Sci.* **43**, 3704–3712.

[bb23] Billinge, S. J. L. & Levin, I. (2005). *Science*, **316**, 561–565. 10.1126/science.1135080 17463280

[bb24] Bordiga, S., Groppo, E., Agostini, G., van Bokhoven, J. A. & Lamberti, C. (2013). *Chem. Rev.* **113**, 1736–1850. 10.1021/cr2000898 23444971

[bb25] Boscherini, F. (2013). *X-ray Absorption Fine Structure in the Study of Semiconductor Heterostructures and Nanostructures* In *Characterization of Semiconductor Heterostructures and Nanostructures*, 2nd ed., edited by C. Lamberti and G. Agostini. Amsterdam: Elsevier.

[bb26] Boscherini, F., de Panfilis, S. & Weissmüller, J. (1998). *Phys. Rev. B*, **57**, 3365–3374.

[bb27] Brouder, C., Ruiz López, M. F., Pettifer, R. F., Benfatto, M. & Natoli, C. R. (1989). *Phys. Rev. B*, **39**, 1488–1500. 10.1103/physrevb.39.1488 9948358

[bb28] Bunker, G. (1983). *Nucl. Instrum. Methods*, **207**, 437–444.

[bb29] Bunker, G. (2010). *Introduction to XAFS: A Practical Guide to X-ray Absorption Fine Structure Spectroscopy.* Cambridge University Press.

[bb30] Cabaret, D., Grand, M. L., Ramos, A., Flank, A.-M., Rossano, S., Galoisy, L., Calas, G. & Ghaleb, D. (2001). *J. Non-Cryst. Solids*, **289**, 1–8.

[bb31] Calvin, S., Riedel, C. J., Carpenter, E. E., Morrison, S. A., Stroud, R. M. & Harris, V. G. (2005). *Phys. Scr.* T**115**, 744–748.

[bb32] Céspedes, E. (2009). *Ferromagnetism in Wide Band Gap Materials: Mn-ZnO and Mn-Si_3_N_4_Thin Films*. PhD thesis. UAM-ICMM, Madrid, Spain.

[bb33] Céspedes, E., Castro, G. R., Jiménez-Villacorta, F., de Andrés, A. & Prieto, C. (2008). *J. Phys. Condens. Matter*, **20**, 095207.

[bb34] Céspedes, E., García-López, J., García-Hernández, M., de Andrés, A. & Prieto, C. (2007). *J. Appl. Phys.* **102**, 033907.

[bb35] Céspedes, E., Sánchez-Marcos, J., García-López, J. & Prieto, C. (2010). *J. Magn. Magn. Mater.* **322**, 1201–1204.

[bb36] Chaboy, J. (2009). *J. Synchrotron Rad.* **16**, 533–544. 10.1107/S0909049509012199 19535869

[bb37] Chaboy, J., Boada, R., Piquer, C., Laguna-Marco, M. A., García-Hernández, M., Carmona, N., Llopis, J., Ruíz-González, M. L., González-Calbet, J., Fernández, J. F. & García, M. A. (2010). *Phys. Rev. B*, **82**, 064411.

[bb38] Chaboy, J., García, L. M., Bartolomé, F., Marcelli, A., Cibin, G., Maruyama, H., Pizzini, S., Rogalev, A., Goedkoop, J. B. & Goulon, J. (1998). *Phys. Rev. B*, **57**, 8424–8429.

[bb39] Chaboy, J., García, L. M., Bartolomé, F., Maruyama, H., Marcelli, A. & Bozukov, L. (1998). *Phys. Rev. B*, **57**, 13386–13389.

[bb40] Chaboy, J., Laguna-Marco, M. A., Piquer, C., Maruyama, H. & Kawamura, N. (2007). *J. Phys. Condens. Matter*, **19**, 436225.

[bb41] Chaboy, J., Marcelli, A. & Bozukov, L. (1995). *J. Phys. Condens. Matter*, **7**, 8197–8210. 10.1103/physrevb.51.9005 9977539

[bb42] Chaboy, J., Muñoz-Páez, A., Carrera, F., Merkling, P. & Sánchez Marcos, E. (2005). *Phys. Rev. B*, **71**, 134208.

[bb43] Chaboy, J. & Quartieri, S. (1995). *Phys. Rev. B*, **52**, 6349–6357. 10.1103/physrevb.52.6349 9981864

[bb44] Chadwick, A. V., Pooley, M. J., Rammutla, K. E., Savin, S. L. P. & Rougier, A. (2003). *J. Phys. Condens. Matter*, **15**, 431.

[bb45] Chang, L., Pattrick, R. A. D., van der Laan, G., Coker, V. S. & Roberts, A. P. (2012). *Can. Mineral.* **50**, 667–674.

[bb46] Chen, C. T., Idzerda, Y. U., Lin, H.-J., Meigs, G., Chaiken, A., Prinz, G. A. & Ho, G. H. (1993). *Phys. Rev. B*, **48**, 642–645. 10.1103/physrevb.48.642 10006830

[bb47] Chen, S., Medhekar, N. V., Garitaonandia, J. & Suzuki, K. (2012). *J. Phys. Chem. C*, **116**, 8541–8547.

[bb48] Chen, S. J., Suzuki, K. & Garitaonandia, J. S. (2009). *Appl. Phys. Lett.* **95**, 172507.

[bb49] Ciatto, G., Di Trolio, A., Fonda, E., Alippi, P., Testa, A. M. & Bonapasta, A. A. (2011). *Phys. Rev. Lett.* **107**, 127206. 10.1103/PhysRevLett.107.127206 22026797

[bb50] Corrias, A., Ennas, G., Mountjoy, E. & Paschina, G. (2000). *Phys. Chem. Chem. Phys.* **2**, 1045–1050.

[bb51] Crespo, P., Litrán, R., Rojas, T. C., Multigner, M., de la Fuente, J. M., Sánchez-López, J. C., García, M. A., Hernando, A., Penadés, S. & Fernández, A. (2004). *Phys. Rev. Lett.* **93**, 087204. 10.1103/PhysRevLett.93.087204 15447222

[bb52] Crocombette, J. P., Pollak, M., Jollet, F., Thromat, N. & Gautier-Soyer, M. (1995). *Phys. Rev. B*, **52**, 3143–3150. 10.1103/physrevb.52.3143 9981430

[bb53] Dalba, G., Fornasini, P. & Rocca, F. (1993). *Phys. Rev. B*, **47**, 8502–8514. 10.1103/physrevb.47.8502 10004887

[bb54] D’Angelo, P., Barone, V., Chillemi, G., Sanna, N., Meyer-Klaucke, W. & Pavel, N. V. (2002). *J. Am. Chem. Soc.* **124**, 1958–1967. 10.1021/ja015685x 11866609

[bb55] D’Angelo, P., Di Nola, A., Filipponi, A., Pavel, N. V. & Roccatano, D. (1994). *J. Chem. Phys.* **100**, 985–994.

[bb56] Demortiere, A., Panissod, P., Pichon, B. P., Pourroy, G., Guillon, D., Donnio, B. & Begin-Colin, S. (2011). *Nanoscale*, **3**, 225–232. 10.1039/c0nr00521e 21060937

[bb57] Díaz-Moreno, S., Koningsberger, D. C. & Muñoz-Páez, A. (1997). *Nucl. Instrum. Methods Phys. Res. B*, **133**, 15–23.

[bb58] Di Cicco, A., D’Amico, F., Zgrablic, G., Principi, E., Gunnella, R., Bencivenga, F., Svetina, C., Masciovecchio, C., Parmigiani, F. & Filipponi, A. (2011). *J. Non-Cryst. Solids*, **357**, 2641–2647.

[bb59] Di Cicco, A. & Trapananti, A. (2005). *J. Phys. Condens. Matter*, **17**, S135–S144.

[bb60] Dietl, T., Ohno, H., Matsukura, F., Cibert, J. & Ferrand, D. (2000). *Science*, **287**, 1019–1022. 10.1126/science.287.5455.1019 10669409

[bb61] Dimakis, N. & Bunker, G. (1998). *Phys. Rev. B*, **58**, 2467–2475.

[bb62] Domínguez-Cañizares, G., Gutiérrez, A., Chaboy, J., Díaz-Fernández, D., Castro, G. & Soriano, L. (2014). *J. Mater. Sci.* **49**, 2773–2780.

[bb63] Dubiel, M., Brunsch, S. & Tröger, L. (2000). *J. Phys. Condens. Matter*, **12**, 4775–4789.

[bb64] Eisenberger, P. & Brown, G. S. (1979). *Solid State Commun.* **29**, 481–484.

[bb65] Ershov, N. V., Ageev, A. L., Vasin, V. V. & Babanov, Y. A. (1981). *Phys. Status Solidi B*, **108**, 103–111.

[bb66] Espinosa, A., Serrano, A., Llavona, A., Jimenez de la Morena, J., Abuin, M., Figuerola, M., Pellegrino, T., Fernández, J. F., García-Hernández, M., Castro, G. R. & García, M. A. (2012). *Meas. Sci. Technol.* **23**, 015602.

[bb67] Farges, F., Lefrère, Y., Rossano, S., Berthereau, A., Calas, G. & Brown, G. E. Jr (2004). *J. Non-Cryst. Solids*, **344**, 176–188.

[bb68] Ferlat, G., Soetens, J.-C., Miguel, A. S. & Bopp, P. A. (2005). *J. Phys. Condens. Matter*, **17**, S145.

[bb69] Fernández-García, M., Martínez-Arias, A., Hanson, J. C. & Rodriguez, J. A. (2004). *Chem. Rev.* **104**, 4063–4104. 10.1021/cr030032f 15352786

[bb70] Filipponi, A. & Di Cicco, A. (1995). *Phys. Rev. B*, **52**, 15135–15149. 10.1103/physrevb.52.15135 9980867

[bb71] Filipponi, A. & Di Cicco, A. (2000). *TASK Q.* **4**, 575–669.

[bb72] Filipponi, A., Di Cicco, A. & Natoli, C. R. (1995). *Phys. Rev. B*, **52**, 15122–15134. 10.1103/physrevb.52.15122 9980866

[bb73] Fisher, C. A. (2004). *Scr. Mater.* **50**, 1045–1049.

[bb74] Fornasini, P. (2001). *J. Phys. Condens. Matter*, **13**, 7859–7872.

[bb75] Frenkel, A. (2007). *Z. Kristallogr.* **22**, 605–611.

[bb76] Frenkel, A. I., Yevick, A., Cooper, C. & Vasic, R. (2011). *Annu. Rev. Anal. Chem.* **4**, 23–39. 10.1146/annurev-anchem-061010-113906 21351882

[bb77] Fujikawa, T. & Miyanaga, T. (1993). *J. Phys. Soc. Jpn*, **62**, 4108–4122.

[bb78] Fukuhara, M. (2003). *Phys. Lett. A*, **313**, 427–430.

[bb79] Funk, T., Deb, A., George, S. J., Wang, H. & Cramer, S. P. (2005). *Coord. Chem. Rev.* **249**, 3–30.

[bb80] Gale, J. D. (1996). *Philos. Mag. B*, **73**, 3–19.

[bb81] Gallego, S., Beltrán, J. I., Cerdá, J. & Muñoz, M. C. (2005). *J. Phys. Condens. Matter*, **17**, L451–L457.

[bb82] Gao, D., Zhang, J., Yang, G., Zhang, J., Shi, Z., Qi, J., Zhang, Z. & Xue, D. (2010). *J. Phys. Chem. C*, **114**, 13477–13481.

[bb83] Garcia, M. A., Merino, J. M., Fernández Pinel, E., Quesada, A., de la Venta, J., Ruíz González, M. L., Castro, G. R., Crespo, P., Llopis, J., González-Calbet, J. M. & Hernando, A. (2007). *Nano Lett.* **7**, 1489–1494. 10.1021/nl070198m 17521211

[bb84] Gereben, O., Jovari, P., Temleitner, L. & Pusztai, L. (2007). *J. Optoelectron. Adv. Mater.* **9**, 3021–3027.

[bb85] Ghosh, M., Biswas, K., Sundaresan, A. & Rao, C. N. R. (2006). *J. Mater. Chem.* **16**, 106–111.

[bb86] Gleiter, H. (1989). *Prog. Mater. Sci.* **33**, 223–315.

[bb87] Gleiter, H. (1995). *Nanostruct. Mater.* **6**, 3–14.

[bb88] Goering, E., Lafkioti, M. & Gold, S. (2006). *Phys. Rev. Lett.* **96**, 039701; discussion 039702. 10.1103/PhysRevLett.96.039701 16486788

[bb89] Goesmann, H. & Feldmann, C. (2010). *Angew. Chem. Int. Ed.* **49**, 1362–1395. 10.1002/anie.200903053 20108287

[bb90] Gota, S., Gautier-Soyer, M. & Sacchi, M. (2000). *Phys. Rev. B*, **62**, 4187–4190.

[bb91] Gouadec, G. & Colomban, P. (2007). *Prog. Cryst. Growth Charact. Mater.* **53**, 1–56.

[bb92] Groppo, E., Prestipino, C., Lamberti, C., Carboni, R., Boscherini, F., Luches, P., Valeri, S. & D’Addato, S. (2004). *Phys. Rev. B*, **70**, 165408.

[bb93] Groppo, E., Prestipino, C., Lamberti, C., Luches, P., Giovanardi, C. & Boscherini, F. (2003). *J. Phys. Chem. B*, **107**, 4597–4606. 10.1103/PhysRevLett.91.046101 12906677

[bb94] Guglieri, C., Céspedes, E., Espinosa, A., Laguna-Marco, M. A., Carmona, N., Takeda, Y., Okane, T., Nakamura, T., García-Hernández, M., García, M. A. & Chaboy, J. (2014). *Adv. Funct. Mater.* **24**, 2094–2100.

[bb95] Guglieri, C., Céspedes, E., Prieto, C. & Chaboy, J. (2011). *J. Phys. Condens. Matter*, **23**, 206006. 10.1088/0953-8984/23/20/206006 21540506

[bb96] Guglieri, C. & Chaboy, J. (2010). *J. Phys. Chem. C*, **114**, 19629–19634.

[bb97] Guglieri, C. & Chaboy, J. (2014). *J. Phys. Chem. C.* In the press.

[bb98] Guglieri, C., Espinosa, A., Carmona, N., Laguna-Marco, M. A., Céspedes, E., Ruíz-González, M. L., González-Calbet, M., García-Hernández, M., García, M. A. & Chaboy, J. (2013). *J. Phys. Chem. C*, **117**, 12199–12209.

[bb99] Guglieri, C., Laguna-Marco, M. A., García, M. A., Carmona, N., Céspedes, E., García-Hernández, M., Espinosa, A. & Chaboy, J. (2012). *J. Phys. Chem. C*, **116**, 6608–6614.

[bb100] Gurman, S. J., Binsted, N. & Ross, I. (1984). *J. Phys. C Solid State Phys.* **17**, 143–151.

[bb101] Gurman, S. J., Binsted, N. & Ross, I. (1986). *J. Phys. C Solid State Phys.* **19**, 1845–1861.

[bb102] Gutiérrez, A., Domínguez-Cañizares, G., Jiménez, J. A., Preda, I., Díaz-Fernández, D., Jiménez-Villacorta, F., Castro, G., Chaboy, J. & Soriano, L. (2013). *Appl. Surf. Sci.* **276**, 832–837.

[bb103] Gutiérrez, L., Lázaro, F. J., Abadía, A. R., Romero, M. S., Quintana, C., Puerto Morales, M., Patiño, C. & Arranz, R. (2006). *J. Inorg. Biochem.* **100**, 1790–1799. 10.1016/j.jinorgbio.2006.06.010 16899298

[bb104] Guttmann, P., Bittencourt, C., Rehbein, S., Umek, P., Ke, X., Tendeloo, G. V., Ewels, C. P. & Schneider, G. (2012). *Nat. Photon.* **6**, 25–29.

[bb105] Halasyamani, P. S. & Poeppelmeier, K. R. (1998). *Chem. Mater.* **10**, 2753–2769.

[bb106] Hattori, Y., Konishi, T. & Kaneko, K. (2002). *Chem. Phys. Lett.* **355**, 37–42.

[bb107] Hong, N. H., Sakai, J. & Brizé, V. (2007). *J. Phys. Condens. Matter*, **19**, 036219.

[bb108] Hsu, H. S., Huang, J. C. A., Huang, Y. H., Liao, Y. F., Lin, M. Z., Lee, C. H., Lee, J. F., Chen, S. F., Lai, L. Y. & Liu, C. P. (2006). *Appl. Phys. Lett.* **88**, 242507.

[bb109] Huang, D. J., Chang, C. F., Jeng, H. T., Guo, G. Y., Lin, H. J., Wu, W. B., Ku, H. C., Fujimori, A., Takahashi, Y. & Chen, C. T. (2004). *Phys. Rev. Lett.* **93**, 077204. 10.1103/PhysRevLett.93.077204 15324271

[bb110] Huang, T.-C., Wang, M.-T., Sheu, H.-S. & Hsieh, W.-F. (2007). *J. Phys. Condens. Matter*, **19**, 476212.

[bb111] Jang, W. L., Lu, Y. M., Hwang, W. S., Dong, C. L., Hsieh, P. H., Chen, C. L., Chan, T. S. & Lee, J. F. (2011). *Europhys. Lett.* **96**, 37009.

[bb112] Jang, W.-L., Lu, Y.-M., Hwang, W.-S., Hsiung, T.-L. & Wang, H. P. (2009). *Appl. Phys. Lett.* **94**, 062103.

[bb113] Kalinko, A. & Kuzmin, A. (2011). *J. Non-Cryst. Solids*, **357**, 2595–2599.

[bb114] Kalinko, A., Kuzmin, A. & Evarestov, R. A. (2009). *Solid State Commun.* **149**, 425–428.

[bb115] Kim, D. H., Lee, H. J., Kim, G., Koo, Y. S., Jung, J. H., Shin, H. J., Kim, J.-Y. & Kang, J.-S. (2009). *Phys. Rev. B*, **79**, 033402.

[bb116] Kohmoto, O., Nakagawa, H., Isagawa, Y. & Chayahara, A. (2001). *J. Magn. Magn. Mater.* **226**, 1629–1630.

[bb117] Krayzman, V. & Levin, I. (2010). *J. Phys. Condens. Matter*, **22**, 404201. 10.1088/0953-8984/22/40/404201 21386562

[bb118] Krayzman, V., Levin, I., Woicik, J. C., Proffen, Th., Vanderah, T. A. & Tucker, M. G. (2009). *J. Appl. Cryst.* **42**, 867–877.

[bb119] Kunz, M. & Brown, I. (1995). *J. Solid State Chem.* **115**, 395–406.

[bb120] Kuzmin, A. & Evarestov, R. A. (2009). *J. Phys. Condens. Matter*, **21**, 055401. 10.1088/0953-8984/21/5/055401 21817299

[bb121] Kuzmin, A., Kalinko, A. & Evarestov, R. A. (2013). *Acta Mater.* **61**, 371–378.

[bb122] Kuzmin, A., Larcheri, S. & Rocca, F. (2007). *J. Phys. Conf. Ser.* **93**, 012045.

[bb123] Kuzmin, A. & Purans, J. (1993). *J. Phys. Condens. Matter*, **5**, 9423–9430.

[bb124] Kuzmin, A. & Purans, J. (2000). *J. Phys. Condens. Matter*, **12**, 1959–1970.

[bb125] Kuzmin, A., Purans, J. & Rodionov, A. (1997). *J. Phys. Condens. Matter*, **9**, 6979–6993.

[bb126] Kuznetsov, A. Y., Machado, R., Gomes, L. S., Achete, C. A., Swamy, V., Muddle, B. C. & Prakapenka, V. (2009). *Appl. Phys. Lett.* **94**, 193117.

[bb127] Laan, G. van der & Kirkman, I. W. (1992). *J. Phys. Condens. Matter*, **4**, 4189–4204.

[bb128] Laan, G. van der, Thole, B. T., Sawatzky, G. A., Goedkoop, J. B., Fuggle, J. C., Esteva, J.-M., Karnatak, R., Remeika, J. P. & Dabkowska, H. A. (1986). *Phys. Rev. B*, **34**, 6529–6531. 10.1103/physrevb.34.6529 9940542

[bb129] Laguna-Marco, M. A., Chaboy, J. & Piquer, C. (2008). *Phys. Rev. B*, **77**, 125132.

[bb130] Laguna-Marco, M. A., Chaboy, J., Piquer, C., Maruyama, H., Ishimatsu, N., Kawamura, N., Takagaki, M. & Suzuki, M. (2005). *Phys. Rev. B*, **72**, 052412.

[bb131] Laguna-Marco, M. A., Piquer, C. & Chaboy, J. (2009). *Phys. Rev. B*, **80**, 144419.

[bb132] Lamberti, C. (2004). *Surf. Sci. Rep.* **53**, 1–197.

[bb133] Lamberti, C., Groppo, E., Prestipino, C., Casassa, S., Ferrari, A. M., Pisani, C., Giovanardi, C., Luches, P., Valeri, S. & Boscherini, F. (2003). *Phys. Rev. Lett.* **91**, 046101. 10.1103/PhysRevLett.91.046101 12906677

[bb134] Larcheri, S., Rocca, F., Jandard, F., Pailharey, D., Graziola, R., Kuzmin, A. & Purans, J. (2008). *Rev. Sci. Instrum.* **79**, 013702. 10.1063/1.2827485 18248034

[bb135] Lee, P. A., Citrin, P. H., Eisenberger, P. & Kincaid, B. M. (1981). *Rev. Mod. Phys.* **53**, 769–806.

[bb136] Lee, P. A. & Pendry, J. B. (1975). *Phys. Rev. B*, **11**, 2795–2811.

[bb137] Levin, I., Krayzman, V. & Woicik, J. C. (2014). *Phys. Rev. B*, **89**, 024106.

[bb138] Li, L., Chen, L., Qihe, R. & Li, G. (2006). *Appl. Phys. Lett.* **89**, 134102.

[bb140] Li, L., Su, Y. & Li, G. (2007). *Appl. Phys. Lett.* **90**, 054105.

[bb139] Li, Z., Dervishi, E., Saini, V., Zheng, L., Yan, W., Wei, S., Xu, Y. & Biris, A. S. (2010). *Particle Sci. Technol.* **28**, 95–131.

[bb141] Liu, E.-Z. L. & Jiang, J. Z. (2010). *J. Appl. Phys.* **107**, 023909.

[bb142] Luches, P., D’Addato, S., Valeri, S., Groppo, E., Prestipino, C., Lamberti, C. & Boscherini, F. (2004). *Phys. Rev. B*, **69**, 045412. 10.1103/PhysRevLett.91.046101 12906677

[bb143] Luches, P., Groppo, E., D’Addato, S., Lamberti, C., Prestipino, C., Valeri, S. & Boscherini, F. (2004). *Surf. Sci.* **566–568**, 84–88.

[bb144] Luches, P., Groppo, E., Prestipino, C., Lamberti, C., Giovanardi, C. & Boscherini, F. (2003). *Nucl. Instrum. Methods Phys. Res. B*, **200**, 371–375.

[bb146] Ma, Q., Prater, J. T., Sudakar, C., Rosenberg, R. A. & Narayan, J. (2012). *J. Phys. Condens. Matter*, **24**, 306002. 10.1088/0953-8984/24/30/306002 22763657

[bb145] Ma, Y. W., Ding, J., Qi, D. C., Yi, J. B., Fan, H. M., Gong, H., Wee, A. T. S. & Rusydi, A. (2009). *Appl. Phys. Lett.* **95**, 072501.

[bb147] Makhlouf, S. A., Kassem, M. A. & Abdel-Rahim, M. A. (2009). *J. Mater. Sci.* **44**, 3438–3444.

[bb148] Mandal, S., Banerjee, S. & Menon, K. S. R. (2009). *Phys. Rev. B*, **80**, 214420.

[bb149] Martínez-Criado, G., Borfecchia, E., Mino, L. & Lamberti, C. (2013). *Micro- and Nano-X-ray Beams* In *Characterization of Semi­conductor Heterostructures and Nanostructures*, 2nd ed., edited by C. Lamberti and G. Agostini. Amsterdam: Elsevier.

[bb150] Martínez-Criado, G., Homs, A., Alén, B., Sans, J. A., Segura-Ruiz, J., Molina-Sánchez, A., Susini, J., Yoo, J. & Yi, G. C. (2012). *Nano Lett.* **12**, 5829–5834. 10.1021/nl303178u 23030721

[bb151] Matsumoto, Y., Murakami, M., Shono, T., Hasegawa, T., Fukumura, T., Kawasaki, M., Ahmet, P., Chikyow, T., Koshihara, S. & Koinuma, H. (2001). *Science*, **291**, 854–856. 10.1126/science.1056186 11228146

[bb152] McGreevy, R. L. (2001). *J. Phys. Condens. Matter*, **13**, R877–R913.

[bb153] McGreevy, R. L. & Pusztai, L. (1988). *Mol. Simul.* **1**, 359–367.

[bb154] Meneses, C. T., Flores, W. H. & Sasaki, J. M. (2007). *Chem. Mater.* **19**, 1024–1027.

[bb155] Merkling, P. J., Muñoz Páez, A., Pappalardo, R. R. & Sánchez Marcos, E. (2001). *Phys. Rev. B*, **64**, 092201.

[bb156] Metropolis, N., Rosenbluth, A. W., Rosenbluth, M. N., Teller, A. H. & Teller, E. (1953). *J. Chem. Phys.* **21**, 1087–1092.

[bb157] Mino, L., Agostini, G., Borfecchia, E., Gianolio, D., Piovano, A., Gallo, E. & Lamberti, C. (2013). *J. Phys. D Appl. Phys.* **46**, 423001.

[bb158] Modrow, H. (2004). *Appl. Spectrosc. Rev.* **39**, 183–290.

[bb159] Morales, M. P., Veintemillas-Verdaguer, S., Montero, M. I., Serna, C. J., Roig, A., Casas, L., Martínez, B. & Sandiumenge, F. (1999). *Chem. Mater.* **11**, 3058–3064.

[bb160] Natoli, C. R., Misemer, D. R., Doniach, S. & Kutzler, F. W. (1980). *Phys. Rev. A*, **22**, 1104–1108.

[bb161] O’Day, P. A., Rivera, N. Jr, Root, R. & Carroll, S. A. (2004). *Am. Mineral.* **89**, 572–585.

[bb162] Oguz Er, A., Chen, J. & Rentzepis, P. M. (2012). *J. Appl. Phys.* **112**, 031101.

[bb163] Ohno, H. (1998). *Science*, **281**, 951–955. 10.1126/science.281.5379.951 9703503

[bb164] Okamoto, Y. (2004). *Nucl. Instrum. Methods Phys. Res. A*, **526**, 572–583.

[bb165] Okudera, H., Yoshiasa, A., Murai, K., Okube, M., Takeda, T. & Kikkawa, S. (2012). *J. Mineral. Petrol. Sci.* **107**, 127–132.

[bb166] Park, J., An, K., Hwang, Y., Park, J. G., Noh, H. J., Kim, J. Y., Park, J. H., Hwang, N. M. & Hyeon, T. (2004). *Nat. Mater.* **3**, 891–895. 10.1038/nmat1251 15568032

[bb167] Patterson, C. H. (2006). *Phys. Rev. B*, **74**, 144432.

[bb168] Pellegrin, E., Hagelstein, M., Doyle, S., Moser, H. O., Fuchs, J., Vollath, D., Schuppler, S., James, M. A., Saxena, S. S., Niesen, L., Rogojanu, O., Sawatzky, G. A., Ferrero, C., Borowski, M., Tjernberg, O. & Brookes, N. B. (1999). *Phys. Status Solidi B*, **215**, 797–801.

[bb169] Pellicer-Porres, J., Segura, A., Sánchez-Royo, J. F., Sans, J. A., Itié, J. P., Flanck, A. M., Lagarde, P. & Polian, A. (2006). *Appl. Phys. Lett.* **89**, 231904.

[bb170] Pérez, N., Bartolomé, F., García, L. M., Bartolomé, J., Morales, M. P., Serna, C. J., Labarta, A. & Batlle, X. (2009). *Appl. Phys. Lett.* **94**, 093108.

[bb171] Pettifer, R. F., Mathon, O., Pascarelli, S., Cooke, M. D. & Gibbs, M. R. (2007). *Nature (London)*, **435**, 78–81. 10.1038/nature03516 15875018

[bb172] Pham, V. T., Tavernelli, I., Milne, C. J., van der Veen, R. M., D’Angelo, P., Bressler, C. & Chergui, M. (2010). *Chem. Phys.* **371**, 24–29.

[bb173] Piquer, C., Roca, A. G., Laguna-Marco, M. A., Boada, R., Guglieri, C. & Chaboy, J. (2014). *J. Phys. Chem. C*, **118**, 1332–1346.

[bb174] Poiarkova, A. V. & Rehr, J. J. (1999). *Phys. Rev. B*, **59**, 948–957.

[bb175] Price, S. W. T., Zonias, N., Skylaris, C.-K., Hyde, T. I., Ravel, B. & Russell, A. E. (2012). *Phys. Rev. B*, **85**, 075439.

[bb176] Purans, J., Afify, N. D., Dalba, G., Grisenti, R., De Panfilis, S., Kuzmin, A., Ozhogin, V. I., Rocca, F., Sanson, A., Tiutiunnikov, S. I. & Fornasini, P. (2008). *Phys. Rev. Lett.* **100**, 055901. 10.1103/PhysRevLett.100.055901 18352392

[bb177] Qi, B., Perez, I., Ansari, P. H., Lu, F. & Croft, M. (1987). *Phys. Rev. B*, **36**, 2972–2975. 10.1103/physrevb.36.2972 9943203

[bb178] Rao, C. & Biswas, K. (2009). *Annu. Rev. Anal. Chem.* **2**, 435–462. 10.1146/annurev-anchem-060908-155236 20636070

[bb179] Rehr, J. J. & Albers, R. C. (2000). *Rev. Mod. Phys.* **72**, 621–654.

[bb180] Rehr, J. J., Kas, J. J., Prange, M. P., Sorini, A. P., Takimoto, Y. & Vila, F. (2009). *C. R. Phys.* **10**, 548–559.

[bb181] Rehr, J. J., Kas, J. J., Vila, F. D., Prange, M. P. & Jorissen, K. (2010). *Phys. Chem. Chem. Phys.* **12**, 5503–5513. 10.1039/b926434e 20445945

[bb182] Ruiz-Lopez, M., Loos, M., Goulon, J., Benfatto, M. & Natoli, C. (1988). *Chem. Phys.* **121**, 419–437.

[bb183] Sanchez, N., Gallego, S. & Muñoz, M. C. (2008). *Phys. Rev. Lett.* **101**, 067206. 10.1103/PhysRevLett.101.067206 18764499

[bb184] Santoyo Salazar, J., Perez, L., de Abril, O., Truong Phuoc, L., Ihiawakrim, D., Vazquez, M., Greneche, J.-M., Begin-Colin, S. & Pourroy, G. (2011). *Chem. Mater.* **23**, 1379–1386.

[bb185] Sato, K. & Katayama-Yoshida, H. (2001). *Physica E*, **10**, 251–255.

[bb186] Sato, H., Minami, T., Takata, S. & Yamada, T. (1993). *Thin Solid Films*, **236**, 27–31.

[bb187] Sayers, D. E. & Bunker, G. (1988). *X-ray Absorption: Principles, Applications, Techniques of EXAFS, SEXAFS, and XANES.* New York: Wiley.

[bb188] Schoenhalz, A. L., Arantes, J. T., Fazzio, A. & Dalpian, G. M. (2009). *Appl. Phys. Lett.* **94**, 162503.

[bb189] Schütz, G., Wagner, W., Wilhelm, W., Kienle, P., Zeller, R., Frahm, R. & Materlik, G. (1987). *Phys. Rev. Lett.* **58**, 737–740. 10.1103/PhysRevLett.58.737 10035022

[bb190] Sevillano, E., Meuth, H. & Rehr, J. J. (1979). *Phys. Rev. B*, **20**, 4908–4911.

[bb191] Smolentsev, G., Soldatov, A. V. & Feiters, M. C. (2007). *Phys. Rev. B*, **75**, 144106.

[bb192] Smolentsev, N., Soldatov, A. V., Smolentsev, G. & Wei, S. Q. (2009). *Solid State Commun.* **149**, 1803–1806.

[bb193] Spezia, R., Duvail, M., Vitorge, P., Cartailler, T., Tortajada, J., Chillemi, G., D’Angelo, P. & Gaigeot, M. P. (2006). *J. Phys. Chem. A*, **110**, 13081–13088. 10.1021/jp064688z 17134169

[bb194] Stern, E. A., Siegel, R. W., Newville, M., Sanders, P. G. & Haskel, D. (1995). *Phys. Rev. Lett.* **75**, 3874–3877. 10.1103/PhysRevLett.75.3874 10059753

[bb195] Stöhr, J. (1999). *J. Magn. Magn. Mater.* **200**, 470–497.

[bb196] Sun, C. Q. (2007). *Prog. Solid State Chem.* **35**, 1–159.

[bb197] Tartaj, P. (2006). *Curr. Nanosci.* **2**, 43–53.

[bb198] Teo, B. K. (1986). *EXAFS: Basic Principles and Data Analysis.* Berlin: Springer.

[bb199] Timoshenko, J., Anspoks, A., Kalinko, A. & Kuzmin, A. (2014*a*). *Phys. Scr.* **89**, 044006.

[bb200] Timoshenko, J., Anspoks, A., Kalinko, A. & Kuzmin, A. (2014*b*). *Acta Mater.* **79**, 194–202.

[bb201] Timoshenko, J., Kuzmin, A. & Purans, J. (2014). *J. Phys. Condens. Matter*, **26**, 055401. 10.1088/0953-8984/26/5/055401 24440877

[bb202] Tomić, S., Searle, B., Wander, A., Harrison, N., Dent, A., Mosselmans, J. & Inglesfield, J. (2005). *New Tools for the Analysis of EXAFS: The DL EXCURV Package.* CCLRC Technical Report DL-TR-2005-001. Daresbury, UK: CCLRC.

[bb203] Tsunekawa, S., Sahara, R., Kawazoe, Y. & Ishikawa, K. (1999). *Appl. Surf. Sci.* **152**, 53–56.

[bb204] Turney, J. E., McGaughey, A. J. H. & Amon, C. H. (2009). *Phys. Rev. B*, **79**, 224305.

[bb205] Vaccari, M. & Fornasini, P. (2006). *J. Synchrotron Rad.* **13**, 321–325. 10.1107/S0909049506018504 16799223

[bb206] Vila, F. D., Rehr, J. J., Rossner, H. H. & Krappe, H. J. (2007). *Phys. Rev. B*, **76**, 014301.

[bb207] Wang, H.-B., Ma, F., Li, Q.-Q., Dong, C.-Z., Ma, D.-Y., Wang, H.-T. & Xu, K.-W. (2013). *Nanoscale*, **5**, 2857–2863. 10.1039/c3nr33584d 23443575

[bb208] Wang, Q., Sun, Q., Chen, G., Kawazoe, Y. & Jena, P. (2008). *Phys. Rev. B*, **77**, 205411.

[bb209] Wang, H.-C., Wei, Y.-L., Yang, Y.-W. & Lee, J.-F. (2005). *J. Electron Spectrosc. Relat. Phenom.* **144–147**, 817–819.

[bb210] Waychunas, G. A., Apted, M. J. & Brown, G. E. Jr (1983). *Phys. Chem. Miner.* **10**, 1–9.

[bb211] Wilke, M., Farges, F., Pettit, P.-M., Brown, G. E. Jr & Martins, F. (2001). *Am. Mineral.* **86**, 714–730.

[bb212] Winterer, M. (2000). *J. Appl. Phys.* **88**, 5635–5644.

[bb213] Xing, G. Z., Wang, D. D., Cheng, C.-J., He, M., Li, S. & Wu, T. (2013). *Appl. Phys. Lett.* **103**, 022402.

[bb214] Yamamoto, Y., Miura, T., Suzuki, M., Kawamura, N., Miyagawa, H., Nakamura, T., Kobayashi, K., Teranishi, T. & Hori, H. (2004). *Phys. Rev. Lett.* **93**, 116801. 10.1103/PhysRevLett.93.116801 15447364

[bb215] Yamasaki, A., Kobori, H., Osawa, H., Nakamura, T. & Sugimura, A. (2009). *J. Phys. Confer. Ser.* **150**, 042235.

[bb216] Yan, W., Sun, Z., Liu, Q., Li, Z., Pan, Z., Wang, J. & Wei, S. (2007). *Appl. Phys. Lett.* **91**, 062113.

[bb217] Yancey, D. F., Chill, S. T., Zhang, L., Frenkel, A. I., Henkelman, G. & Crooks, R. M. (2013). *Chem. Sci.* **4**, 2912–2921.

[bb218] Yano, J. & Yachandra, V. K. (2009). *Photosynth. Res.* **102**, 241–254. 10.1007/s11120-009-9473-8 PMC2777224 19653117

[bb219] Yu, J., Rosso, K. M. & Bruemmer, S. M. (2012). *J. Phys. Chem. C*, **116**, 1948–1954.

[bb220] Zabinsky, S. I., Rehr, J. J., Ankudinov, A., Albers, R. C. & Eller, M. J. (1995). *Phys. Rev. B*, **52**, 2995–3009. 10.1103/physrevb.52.2995 9981373

[bb221] Zhang, S., Zhang, L., Li, H., Li, J., Jiang, Z., Chu, W., Huang, Y., Wang, J. & Wu, Z. (2010). *J. Synchrotron Rad.* **17**, 600–605. 10.1107/S0909049510020303 20724781

[bb222] Zheng, X. G., Kubozono, H., Yamada, H., Kato, K., Ishiwata, Y. & Xu, C. N. (2008). *Nat. Nanotechnol.* **3**, 724–726. 10.1038/nnano.2008.309 19057591

